# Text classification algorithm of tourist attractions subcategories with modified TF-IDF and Word2Vec

**DOI:** 10.1371/journal.pone.0305095

**Published:** 2024-10-18

**Authors:** Lu Xiao, Qiaoxing Li, Qian Ma, Jiasheng Shen, Yong Yang, Danyang Li

**Affiliations:** 1 School of Management, Guizhou University, Guiyang, China; 2 School of Tourism Management, Guizhou University of Commerce, Guiyang, China; 3 The research center of development strategy in Karst Region, Guizhou University, Guiyang, China; 4 Guiyang QianYanJiuYi Cultural & Information Consulting Co., Ltd., Guiyang, China; 5 Research and Development Department, Shenzhen Costar Software Co., Ltd., Shenzhen, China; 6 Colleges of Big Data and Information Engineering, Guizhou University, Guiyang, China; University of Sargodha, PAKISTAN

## Abstract

Text classification, as an important research area of text mining, can quickly and effectively extract valuable information to address the challenges of organizing and managing large-scale text data in the era of big data. Currently, the related research on text classification tends to focus on the application in fields such as information filtering, information retrieval, public opinion monitoring, and library and information, with few studies applying text classification methods to the field of tourist attractions. In light of this, a corpus of tourist attraction description texts is constructed using web crawler technology in this paper. We propose a novel text representation method that combines Word2Vec word embeddings with TF-IDF-CRF-POS weighting, optimizing traditional TF-IDF by incorporating total relative term frequency, category discriminability, and part-of-speech information. Subsequently, the proposed algorithm respectively combines seven commonly used classifiers (DT, SVM, LR, NB, MLP, RF, and KNN), known for their good performance, to achieve multi-class text classification for six subcategories of national A-level tourist attractions. The effectiveness and superiority of this algorithm are validated by comparing the overall performance, specific category performance, and model stability against several commonly used text representation methods. The results demonstrate that the newly proposed algorithm achieves higher accuracy and F1-measure on this type of professional dataset, and even outperforms the high-performance BERT classification model currently favored by the industry. Acc, marco-F1, and mirco-F1 values are respectively 2.29%, 5.55%, and 2.90% higher. Moreover, the algorithm can identify rare categories in the imbalanced dataset and exhibit better stability across datasets of different sizes. Overall, the algorithm presented in this paper exhibits superior classification performance and robustness. In addition, the conclusions obtained by the predicted value and the true value are consistent, indicating that this algorithm is practical. The professional domain text dataset used in this paper poses higher challenges due to its complexity (uneven text length, relatively imbalanced categories), and a high degree of similarity between categories. However, this proposed algorithm can efficiently implement the classification of multiple subcategories of this type of text set, which is a beneficial exploration of the application research of complex Chinese text datasets in specific fields, and provides a useful reference for the vector expression and classification of text datasets with similar content.

## 1. Introduction

In recent years, with the rapid progress of the Internet, the volume of electronic information has been constantly expanding. According to the Digital China Development Report (2021) released by the Cyberspace Administration of China, China’s data production has grown from 2.3 ZB in 2017 to 6.6 ZB in 2021, representing a global share of 9.9% (Cyberspace Administration of China. The Digital China Development Report (2021) [EB/OL]. (2022-08-02) [2022-9-9]. http://www.cac.gov. cn/2022-08/02/c_1661066515613920.htm). Currently, the volume of data on the global Internet exceeds 66,000 Exabytes, with an immeasurable amount of textual information continuously increasing every day. In the era of big data, quickly and accurately extracting socially and commercially valuable information from these textual data has become a pressing concern for researchers, leading to the growing popularity of text mining in the field of knowledge discovery. As a result, automatic text classification technology, an important component of text mining, has emerged as one of the research hotspots [1].

Automatic text classification (hereinafter referred to as text classification) falls under the category of supervised learning algorithms in artificial intelligence. It aims to build a model that captures the relationship between text feature items and text categories by learning from a labeled text set. This model is then applied to categorize new texts. Text classification techniques can be classified into two types based on different principles: knowledge-based methods and machine learning-based(ML-based) methods [2]. Due to the slow and resource-intensive nature of knowledge base updates, knowledge-based methods are less commonly employed in practical applications. With the continuous development of artificial intelligence technology, ML-based classification methods have rapidly emerged and gained popularity. Because of their strong adaptability and fast classification speed, ML-based methods have become the first choice for automatic text classification. They have shown diverse research outcomes and are widely used in various fields such as information filtering, information retrieval, data organization, public opinion monitoring, sentiment analysis, and personalized recommendation [3–5].

The tourism industry is currently experiencing a boom, with a plethora of travel information available on social media platforms such as Weibo, WeChat, and e-commerce sites. However, there is a limited number of research studies utilizing artificial intelligence in the tourism industry. Most of the existing research focuses on tourism big data mining [6–8]. According to the latest data, China has more than 14,000 national A-level tourist attractions, and the descriptive texts of these attractions exist in an unstructured form. If these attractions are classified manually based on their descriptive texts, it would require a lot of time and cost and would be subject to subjective errors. Furthermore, research on the automatic classification of tourism texts is scarce and mainly concentrated on binary or ternary classification studies, such as sentiment analysis. And there is a lack of research on Chinese text classification for multi-class subcategories. Among them, Chinese text classification is more difficult because it needs to overcome challenges such as word segmentation, semantic complexity, and word order information [9, 10]. In addition, most of the existing results of text classification are based on large-category classification research conducted on public datasets. For example, the THUCNews dataset was used to classify Sina News into 10 parallel categories such as sports, finance, real estate, home furnishings, education, technology, fashion, politics, games, and entertainment [11]. The content of each category’s news text differs significantly.

Considering the above, this paper proposes a text classification model that combines Word2vec word embedding, improved TF-IDF (Term Frequency-Inverse Document Frequency) weighting (TF-IDF-CRF-POS), and a machine-learning classification algorithm. The model is applied to a corpus of tourist attraction description texts obtained through crawling and organizing, to achieve multi-classification of the subcategories of A-level tourist attractions.

The main contribution of this paper is as follows:

1. Innovations in the improved weighting of TF-IDF. On the one hand, the impact of text length on classification is rarely considered in the existing research. However, in practical applications, a feature word often appears more frequently in long texts than in short texts. If the situation is not considered, the classification effect will be affected. On the other hand, although some studies have introduced category factors for improvement, they have overlooked the problem that the predicted samples lack category labels in practical applications during the testing phase. This is not in line with the applied logic, resulting in biased and unreliable test results, making it difficult to apply to actual predictions. Based on improving the above two issues, this paper comprehensively considers several factors that are more important for text classification, including the total relative frequency of feature words, category distribution and discrimination, part of speech of feature words, and contextual semantic relations, to represent text. Compared to the factors considered in the existing research, this proposed method is more comprehensive and more practical for real-world applications.
2. This paper focuses on the classification of subcategories of complex text datasets with similar content, which is more challenging in the field of text classification. Content similarity refers to the resemblance in sentence structures, constituent words, expression patterns, etc., within the description texts of tourist attractions. Complexity is reflected in the uneven length of classified texts, the imbalance of categories, and the fact that the text language is Chinese. And subcategories represent categories with hierarchical relationships, for example, cultural and natural tourist attractions are both subcategories under tourist attractions. Therefore, this paper investigates tourism text classification based on the distinctive information contained in the texts describing tourist attractions. The proposed classification method not only improves the classification efficiency of tourism texts but also efficiently and objectively realizes multi-classification tasks, enriching the application of artificial intelligence in the tourism field. Furthermore, it can be extended to other common practice fields for text classification such as scientific research and education and it is a useful exploration of the application research of complex Chinese text datasets in specific fields.
3. This paper not only focuses on theoretical contributions but also emphasizes the practicality of the method. Currently, many studies only demonstrate the improved effects and performance comparisons of their methods, without a detailed analysis of their feasibility in practical applications. By randomly selecting the data of two provinces, this paper applies its method to forecasting and compares the conclusions drawn from the true values and the predicted values. Through specific case studies, this paper demonstrates how to apply its method to real-world problems and discusses the credibility of its conclusions in practical applications, thereby illustrating the application feasibility of the method.
4. Moreover, due to the shortage of research results using artificial intelligence technology in the tourism industry, there is no a perfect Chinese tourism text corpus and classification dataset in the industry. This paper constructs a text corpus comprising scenic spot descriptions and a classification dataset encompassing different types of tourist attractions. These resources can enrich the fundamental research data in the tourism field and provide convenience for subsequent researchers, thereby promoting the research process of artificial intelligence applications in the tourism industry.

## 2. Related research

Text classification research can be traced back to the 1950’s. Luhn [12] first proposed the use of Term Frequency (TF) statistics as a means of feature selection in 1958. Maron et al. [13] pioneered the probabilistic index model and applied the Bayesian algorithm to text classification, which greatly accelerated the pace of text classification research. In the 1970s, Salton et al. [14] proposed the vector space model. During the 1980s, knowledge engineering became the primary technique for text classification. However, this approach required experts from various fields to provide knowledge to form rules, which means building text classifiers manually. In the 1990s, text classification methods based on statistics and machine learning(ML) gradually emerged. These methods involved machines extracting rules for effective classification from documents and learning these rules to predict classifications. This approach eliminated the need for manual intervention and overcame the limitations of manually constructed classifiers, making text classification more accurate and effective. Over several decades of development, ML-based text classification methods have established a relatively comprehensive theoretical system. They have become the dominant approach and have been applied in many fields, including the automatic classification of web documents, automatic abstracting, and digital libraries, etc. The fundamental work of ML-based text classification generally includes text pre-processing, feature extraction, classifier construction, and evaluation. Among them, feature extraction (that is text representation) plays a crucial role. The quality of text representation directly influences the performance of the classifier. In recent years, many researchers have focused on extracting text features by combining different methods or improving feature extraction techniques.

### 2.1. Related studies on text representation

Word embedding technology is currently a popular and effective text representation method. The early traditional word representation method mainly relied on the Bag-Of-Words(BOW) model, such as discrete representation methods represented by one-hot and TF-IDF [15] representations. However, these methods did not consider the semantics of the words themselves. Consequently, the current mainstream methods for distributed representation, such as Word2vec [16] and Doc2vec [17], have gradually emerged. These methods aim to capture the semantic relations between words and have significantly advanced the field of text representation.

In the existing studies, researchers have explored various aspects of feature representation methods. On the one hand, some studies have compared individual feature representation methods. For instance, several studies showed that Word2vec outperformed TF-IDF. However, Liu et al. [18] concluded that Word2vec and TF-IDF are suitable for the classification recognition of different content. For example, Purpura et al. [19] evaluated the effectiveness of TF-IDF in identifying single-item faked responses. Arruda et al. [20] compared Word2vec and Doc2Vec, and found that the feature representation method based on Word2vec (98.72% accuracy) is far superior to the method based on Doc2Vec (70.8% accuracy) in classification research. Similarly, Wang et al. [21] compared the performance of these three feature representation methods in short text classification, revealing a decreasing accuracy order of TF-IDF, Word2Vec, and Doc2vec. On the other hand, more researchers have been exploring the combination of different feature representation methods in text classification. For example, Zhu et al. [22] discovered that the DF-CHI combined feature extraction method greatly improved the classification performance of classifiers; Seethappana et al. [23] discovered the method that closely combined n-gram feature sets with TF-IDF weighting outperformed other text representation methods in classifying euphemisms. Since Word2Vec captures the semantic relations and TF-IDF reflects the importance of words in the text, the two methods can complement each other. This is why most researchers focus on combining TF-IDF and Word2Vec for text representation, classification, and recognition in various fields, resulting in performance improvement. For example, Mohamed et al. [24] utilized the combination of TF-IDF and Word2Vec to represent text features and applied them to classify the multi-label classification of Holy Quran scriptures, achieving favorable outcomes. Wang et al. [25] proposed a log unsupervised anomaly detection method (LogUAD) based on Word2Vec word vectors and TF-IDF weighted sequence feature vectors and compared it with the LogCluster method, finding that the F1-score of LogUAD can be improved by 67.25%. Peng et al. [26] improved the TF-IDF algorithm to obtain the word contribution degree and combined it with Word2Vec to propose a new text representation method, which demonstrated better classification performance compared to traditional methods, such as TF-IDF, mean Word2Vec and PTF-IDF weighted Word2Vec models.

### 2.2. Related studies on TF-IDF improvement

In response to the shortcomings of the traditional TF-IDF, which does not reflect the importance of feature words well in text representation, many scholars have made improvements based on it, particularly in terms of category discrimination and category distribution. For example, Li et al. [27] introduced category discrimination, location information, and part of speech to improve the TF-IDF model. They combined it with LDA to create a composite weighted model, achieving optimal performance in bibliographic information classification. Chen [28] improved TF-IDF based on intra-class and inter-class distributions, as well as location information. Then, used the improved TF-IDF-ICP method to weight the Word2Vec word vector for text representation and combined it with the improved SPCA-LSTM algorithm to analyze the emotions of rural tourists. The result showed that the performance index was better than that of the existing algorithm. In addition, a few scholars also incorporated other elements, such as location weights, word span weights, word nature, and specificity degree, to improve the TF-IDF model. For instance, Zhang [29] added location weights and word span weights to the traditional TF-IDF keyword weight calculation method and considered multiple feature terms of words to improve the classification effect. Mohammed et al. [30] gradually added part-of-speech and Word2Vec semantic representation to the traditional TF-IDF. Finally, the TFPOS-IDF method and W2V-TFPOSIDF method based on feature improvement were obtained. Experimental verification showed that the W2V-TFPOSIDF method exhibited better classification performance. Yu et al. [31] reconstructed the TF-IDF model by introducing the specificity degree (SD) information, effectively improving the accuracy of patent extraction.

### 2.3. Related studies on text classification in the tourism field

There is limited research on the application of automatic text classification in the tourism field, with a primary focus on sentiment classification of customer group review texts and personalized recommendations. For example, Ray et al. [32] provided word embeddings generated by a pre-trained BERT model, word vectors generated by Word2Vec, TF-IDF values of words as input to the SVM classifier, which achieved an F1-score of 84% and accuracy of 92.36% in sentiment classification of hotel reviews, while also developing a recommendation system based on sentiment analysis. Cheng et al. [33] employed TF-IDF values to select keywords and utilized SnowNLP to calculate the emotion scores of sentences. They categorized emotional responses into positive, negative, and negative of positive, and examined the relationship between emotional responses with tourists’ identity. These studies mainly focused on binary or ternary classification, and there is a significant lack of research on more multiclass automatic classification (relatively greater complexity) of tourism texts in the academic community. However, Wang et al. [34] used information gain to select vocabulary features and applied TF-IDF to assign weights, with the combination of SVM, KNN, DT, LR, and NB classifiers to achieve multi-classification of tourism texts in Minnan culture, Hakka culture, red culture, and ecological culture. Apart from this, limited research has been conducted so far.

In summary, while related research on text classification has yielded positive results in different fields, the application of an improved TF-IDF+Word2Vec text representation model to achieve multi-classification of subcategories in the tourism field has not been explored. Therefore, this paper aims to explore it.

## 3. Models and algorithm process

**Fig 1:**
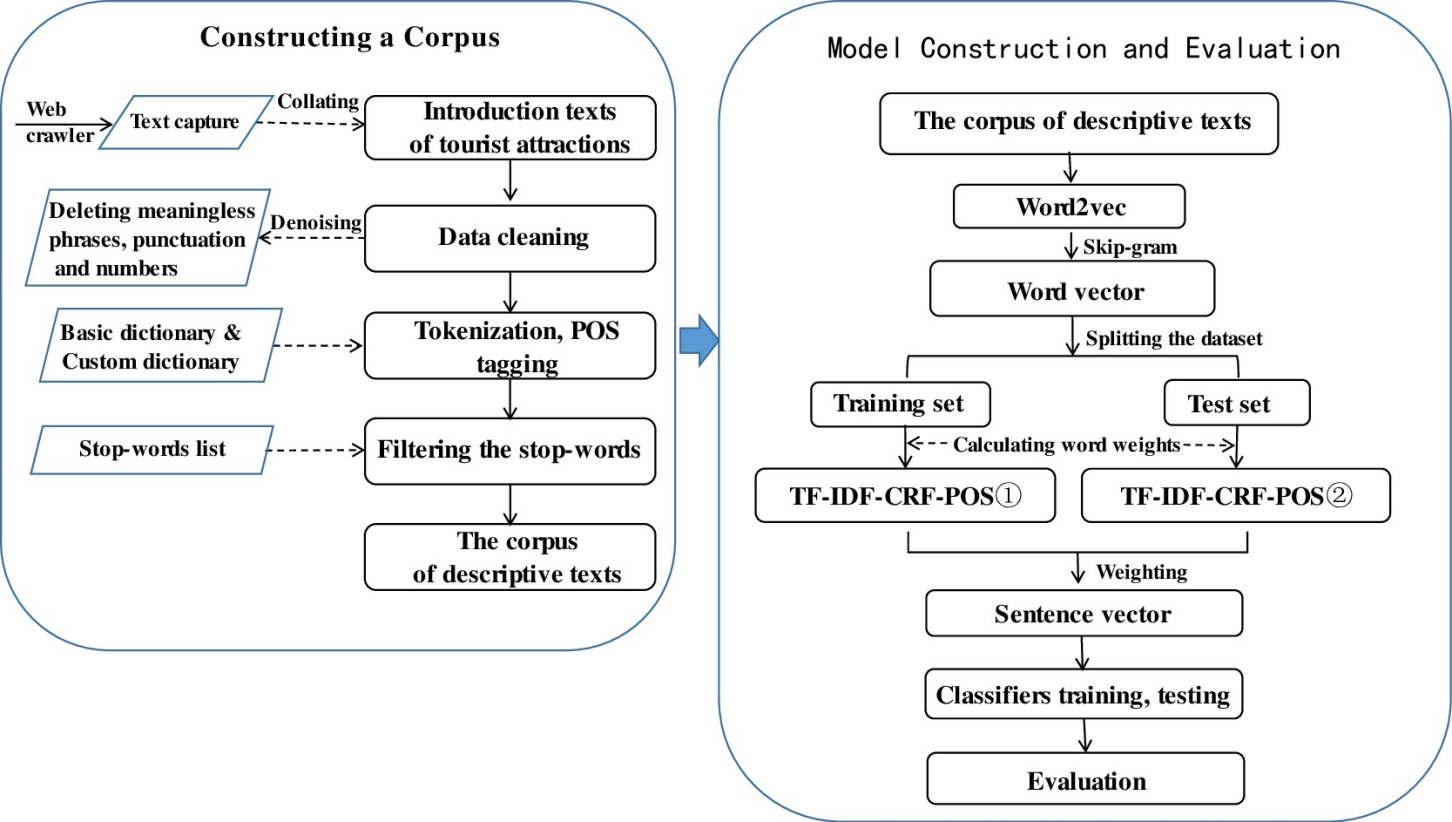
Flowchart of the framework of text classification algorithm for subordinate classes of tourist attractions.

This part explores the text-mining classification algorithm for subcategories of tourist attractions. The algorithm first used the neural network language model of Word2ve to obtain word vectors by training on a large-scale corpus. Next, it utilized an improved formula for TF-IDF to calculate the weight of each word and obtain weighted word vectors. It then constructed a representation matrix of the text vectors. Finally, it fed the text vectors into different classification models to explore the best combination of models for achieving better text classification predictions, and to apply the algorithm to real-world classification tasks. Fig 1 displays the flowchart of the algorithm, including key processes such as data preparation (building corpus), training the Word2Vec word vector, calculating word weight and sentence vectors, as well as training and evaluating the classifiers.

### 3.1. Data preparation

The data preparation for this algorithm includes the following processes.

1. Data acquisition and cleaning
  The data used in this paper was obtained from the official websites of the Department of Culture and Tourism of each province in China, the Maigoo website (https://www.maigoo.com), and the Baidu Encyclopedia (https://baike.baidu.com), etc. The list and introduction texts of national A-level tourist attractions in every province of China were collected through the web crawler that we developed using Python and manual search, which formed the initial text set. To reduce data noise and dimension, and improve the accuracy of the output results, sentences unrelated to the classification task of this paper (such as statements about location, latitude, and longitude of tourist attractions), punctuation marks, and numbers were removed, etc. The initial corpus for this paper was obtained after data cleaning.
2. Text preprocessing
  The corpus must be pre-processed to be transformed into a format that the Word2Vec model can process. The text preprocessing mainly involves Chinese word segmentation, part-of-speech tagging, and stop-word filtering, etc. First, the texts were segmented and tagged for part of speech using the accurate mode of Jieba which is a third-party Python library.

Meanwhile, based on the characteristics of the corpus and the word segmentation results without a custom dictionary, proper nouns such as place names, specialized terms in the field of tourism, as well as some proverbs and idioms that were not successfully segmented were added to the list to create a custom dictionary. This would help to make the segmentation system better suited for tourism applications and achieve more accurate segmentation. Second, the stop words were removed. The stop words list needs to be adjusted according to the actual situation of the text processing task and the segmentation results of the corpus. In this paper, the customized stop word list was generated by combining and removing duplicates of three commonly used Chinese stop word lists: Baidu, Harbin Institute of Technology, and Machine Intelligence Laboratory of Sichuan University. Then the list was expanded to include both Chinese and English terms for number and metric unit (e.g. 米, 千米, m, km^2^, mgL), and some words that caused inaccurate segmentation in the stop word list were corrected. Ultimately, a customized stop words list consisting of 1739 words was created. By using this list to filter out meaningless or noisy words and eliminate single-character words, the final corpus of tourist attraction descriptions can be obtained.

### 3.2. Word vector training

**Fig 2:**
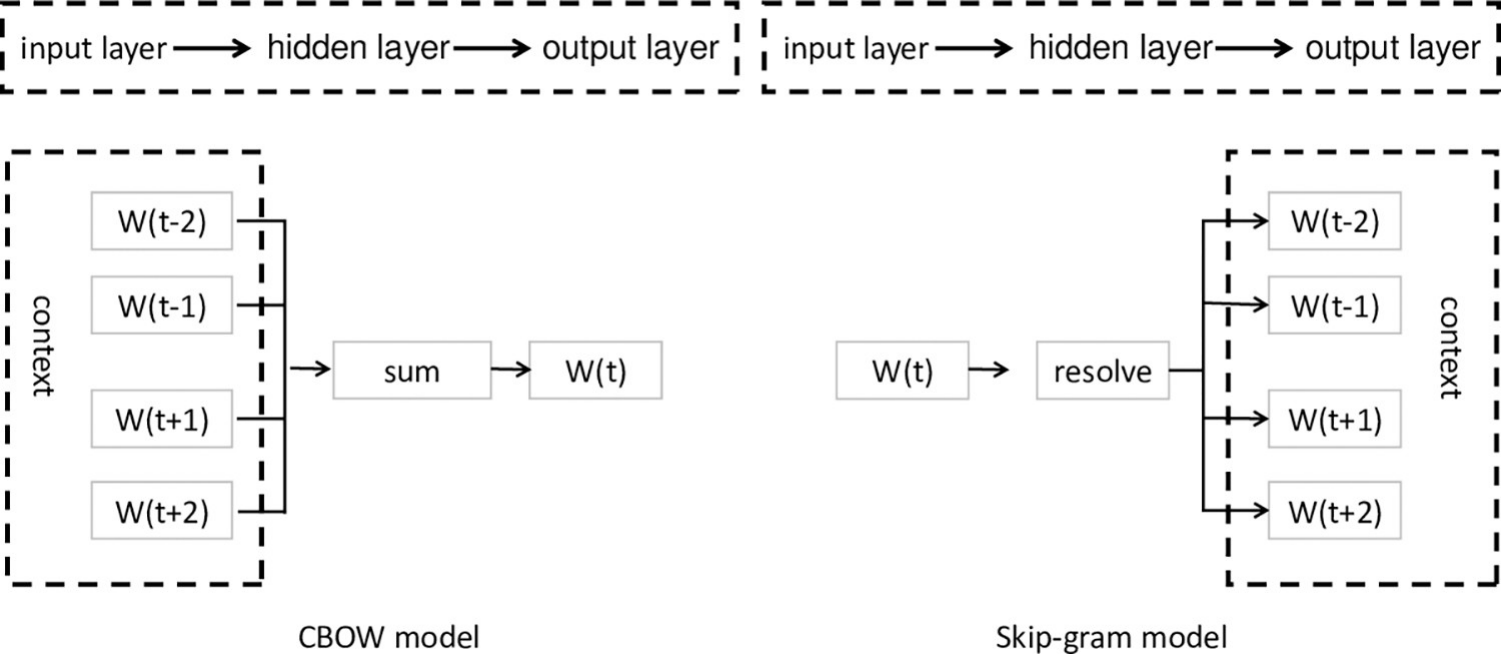
CBOW and skip-gram models [36].

Text classification tasks require solving the issue of text representation. Word vector training is one approach that involves extracting text features to represent text strings as numerical vectors that computers can process. In this paper, the Word2Vec model was used to train the corpus of tourist attraction descriptions, which results in word vectors for all words. The Word2Vec model, proposed by Google in 2013 by Mikolov et al, is currently a mature and widely used word embedding model that can efficiently represent words as m-dimensional real number vectors based on a large-scale unsupervised text corpus. The Word2Vec model consists of three layers of neural networks, namely the input layer, hidden layer, and output layer, including two training models, CBOW and Skip-gram, whose architecture is illustrated in Fig 2. The CBOW predicts the occurrence probability of the current word through the context, i.e., it uses the *k* words before and after the word to predict the current word and *k* is called the context window size. The Skip-Gram, on the contrary, predicts the occurrence probability of contextual words through the current word, i.e., the word is used to predict the *k* words before and after it [35].

**Fig 3:**
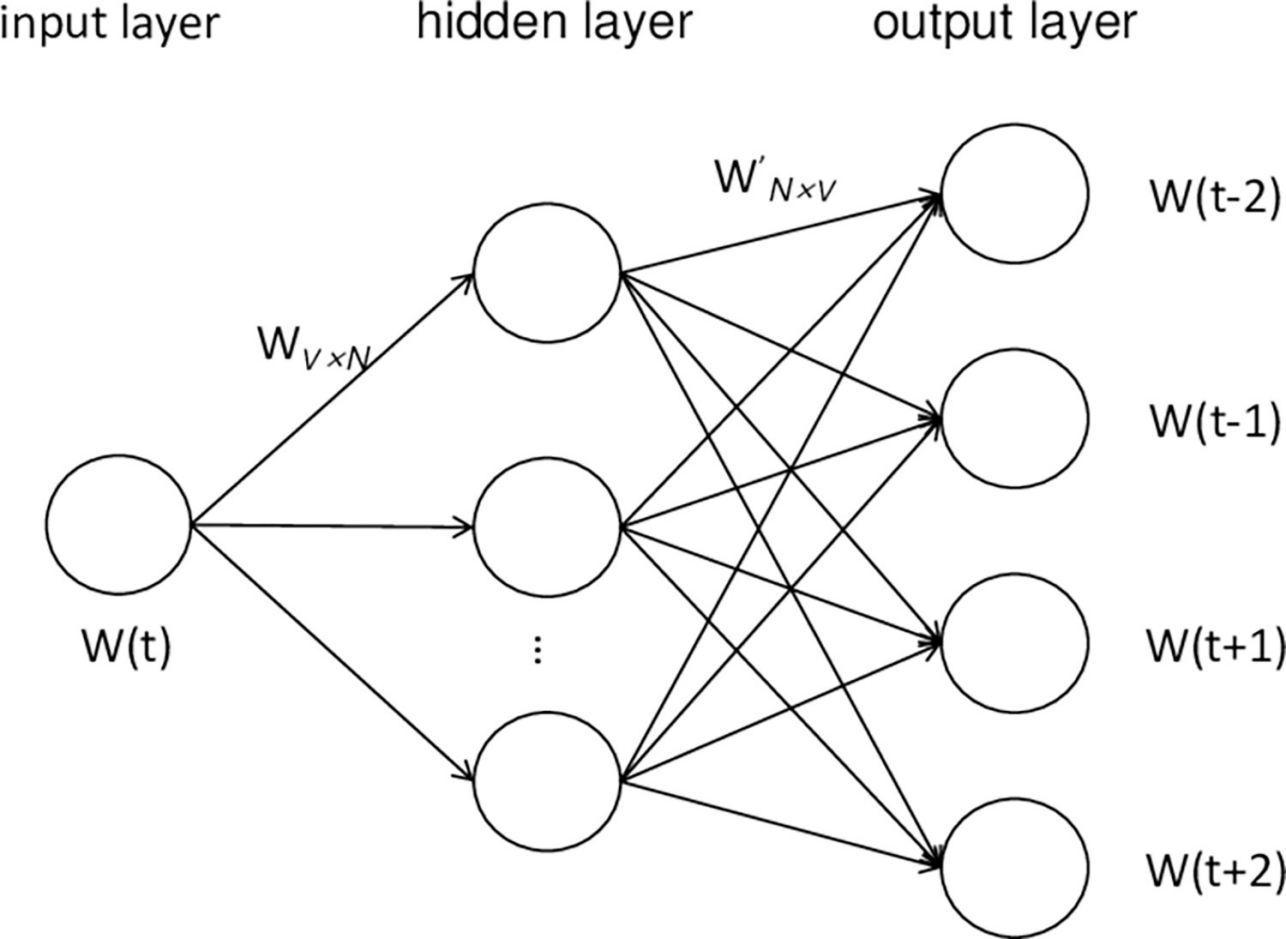
Structure of skip-gram model [37].

The Skip-gram usually takes longer training time compared to the CBOW, but it often achieves better accuracy. Therefore, the Skip-gram is selected to obtain more accurate results in this paper, and its structure is shown in Fig 3. Its objective function is:

$$\max\frac{1}{N}\sum_{n=1}^{N}\left[\sum_{-k\leq j\leq k,j\neq 0}\log p(w_{n+j}|w_{n}\right)]$$ (1)

Where *N* is the number of training words, *k* is the size of the training window, *w*_1_ *w*_2_…, *w*_*N*_ is the sequence of training words, and *P* is the probability.

The main objective of the model is to maximize the average log probability of all words through training. However, the accuracy of the word vectors in Word2Vec also depends on other parameters. Among them, more important parameters such as the word vector dimensionality and the window size (i.e., the number of adjacent words before and after) are included. In addition, the Doc2vec text representation is compared with the feature representation in this paper. The Doc2vec adds one paragraph vector that has the same length as the word vector. This allows the model to directly vectorize the text along with the contextual words, resulting in a sentence or text vector. Doc2vec includes two models, DBOW and DM, which are similar in principle to Skip-gram and CBOW of Word2Vec. Both DBOW and Skip-Gram predict the occurrence probability of the context based on existing words, while both DM and CBOW predict the occurrence probability of the current word through the context [38]. This paper adopts the DBOW of Doc2vec. Although the dimensions of the sentence vector obtained by Doc2vec and the word vector by Word2Vec are the same, they represent two different vector spaces.

### 3.3. Improved model and algorithm for TF-IDF

After preparing the data and training the word vectors, the weights of feature words are obtained by improving the traditional TF-IDF model.

#### First, introduce the traditional TF-IDF

The traditional TF-IDF, proposed by Salton [15], was first applied in the field of information retrieval and subsequently became a widely used method for weighting calculation. This model is used to calculate the importance of a feature word in the entire corpus. The core idea of TF-IDF is that the more frequently a feature word appears in a specific text and the fewer documents of the text set it appears in, the more important the feature word is. It is more representative in that text. When calculating IDF, this model adds 1 to the base of the logarithm to prevent the weight from being negative. The common formula for calculating TF-IDF is as follows:

$$W_{i,j}=tf\times idf=\frac{n_{i,j}}{\sum_{k}n_{k,j}}\times \log(\frac{N}{df_{i}}+1)$$ (2)

Where *tf* is the frequency of the feature word in the text, *idf* is the inverse document frequency of the feature word. It indicates that the fewer documents a feature word appears in, the more important the word is and the larger this value *idf* is. *n*_*i*,*j*_ is the frequency of feature word *i*s in the text *j*, $\sum_{k}n_{k,j}$ is the total number of words in the text *j*, *N* is the total number of documents in the text set, and *df*_*i*_ is the number of documents that contain the word *i* in the text set.

#### Second, discuss the improvement idea of traditional TF-IDF

This paper improves the traditional TF-IDF calculation by considering the total relative word frequency of feature words in the entire text set, the distribution difference between different categories, and the word nature, which were not taken into account in traditional TF-IDF. The aim is to better reflect the importance of feature words in the text and more accurately represent the text to improve the text classification performance. The processes for improving TF-IDF are as follows.

Step 1: Introduce the total relative word frequency. A feature word tends to appear more frequently in a long text than in a short text. If this situation is not distinguished, it will affect the classification performance. When the TF and IDF of two feature words are equal in Eq 2, the importance of the two words to their respective text will be different if the length of the text in which the feature word is located and the total frequency of the two feature words in the whole text set are not equal. Therefore, this paper introduces the relative word frequency of the frequency of the word *i* in a text relative to the total frequency of the word *i* in the text set, and the relative word frequency of the length of this text relative to the total length of the text set. Then the formula of the total relative word frequency of the feature word *i* is as follows:

$$P_{i}=\frac{P(q_{i})}{P(p_{j})\times \lambda }=\frac{\frac{n_{i,j}}{\sum_{N}n_{i,N}}}{\frac{\sum_{k}n_{k,j}}{\sum_{N}(\sum_{k}n_{k,j})}\times \lambda }$$ (3)

Where P(*q*_*i*_) represents the relative frequency of the number of word *i* in text *j* relative to the count of words *i* in all texts, and its value is equal to *n*_*i*,*j*_ divided by the total number $\sum_{N}n_{i,N}$ of word *i*s in all texts. P(*p*_*j*_) denotes the relative frequency of the total number of words in the text *j* relative to the total number of words in all texts, and its value is equal to the total number $\sum_{k}n_{k,j}$ of words in the text *j* divided by the total number $\sum_{N}(\sum_{k}n_{k,j})$ of words in all texts. The constant *λ* is introduced as a regulating factor, which serves to amplify the denominator while avoiding too small denominators, and *λ* is set as a constant 2 through experiments.

Step 2: Improve category differentiation. The importance of inter-class information in text classification tasks is self-evident. To enhance the weight of feature words with high category differentiation, the entire text set is divided into *m* (*m* is the number of categories) subsets according to the category of each text, and each category is considered as a whole. Then the category factor is considered in the inverse document frequency (*idf*), and the original *idf* is replaced by the category-distinguished *idf*_*l*_. At the same time, the denominator is smoothed by adding 1 to avoid the denominator from being 0. The calculation formula for improved IDF is as follows:

$$idf_{l}=\log(\frac{P(o_{i})}{P(n_{i})})=\log(\frac{\frac{df_{i,o}}{N_{o}}}{\frac{df_{i,n}}{N_{n}}+1})$$ (4)

Where P(*o*_*i*_) indicates the probability of the feature word *i* appearing in class *o*. and its value is equal to the ratio of the number *df*_*i*,*o*_ of texts containing the word *i* in class *o* to the total number *N*_*o*_ of texts in this category. P(*n*_*i*_) indicates the probability of the feature word *i* appearing in other classes of the text set (by deleting the texts of the class *o*), and its value is equal to the ratio of the number *df*_*i*,*n*_ of texts containing the word *i* in the other classes to the total number *N*_*n*_ of texts in the other classes.

In Eq 4, P(*o*_*i*_) denotes the concentration of the feature word *i* in the category *o*, while P(*n*_*i*_) denotes the concentration of the feature word *i* in the other categories. The larger the value of P(*o*_*i*_) is and the smaller the value of P(*n*_*i*_) is, the larger the value of P(*o*_*i*_)/P(*n*_*i*_) is, which means that the feature word *i* is mainly distributed in the category *o* and rarely appears in other categories of texts. So the feature word *i* has a stronger classification ability and and is more representative of this category, it should be given higher weight. As the weight of the feature term *i* increases in non-*o* of classes, that is the value of P(*o*_*i*_)/P(*n*_*i*_) becomes smaller, the feature term *i* is less representative for the category *o*, and the weight assigned to it is correspondingly reduced. The improved TF-IDF algorithm can better distinguish the distribution of different feature words compared to the traditional TF-IDF algorithm when the number of text appearing in two feature words is the same, but the distribution of feature words is different between P(*o*_*i*_) and P(*n*_*i*_), resulting in different IDF values. When two terms appear in the same number of texts, P(*o*_*i*_) and P(*n*_*i*_) are different due to the different distribution of the two terms, resulting in different IDF values. Therefore, The improved TF-IDF algorithm can better distinguish the distribution of different feature words compared to the traditional TF-IDF algorithm.

Step 3: Introduce the distinctions of the word nature. Feature words with different parts of speech have different importance in the sentence. In general, nouns (excluding proper nouns such as place names) and verbs are more important than other part-of-speech words, so word nature is taken into account in TF-IDF. In this paper, *pos*_*wi*_ is introduced as a POS tagging weighting value to give different weights to words with different parts of speech. The formula is as follows:

$$pos_{wi}=\{\begin{array}{l} 1.5\;(verb\;or\;noun)\\ 1\;(others) \end{array}$$ (5)

*pos*_*wi*_ takes 1.5 when *i* is a noun or verb, otherwise *pos*_*wi*_ takes 1.

#### Third, determine the final improved model of the traditional TF-IDF

Based on the above improvement of the traditional TF-IDF, this paper integrates Eq 3, Eq 4, and Eq 5 into Eq 2 to obtain the comprehensive improved formula for calculating the weights of feature words *i* in the category *o*, that is called TF-IDF-CRF-POS (Term Frequency-Inverse Document Frequency with Classification and Relative Frequencies-Part of speech) formula. This formula is used to calculate the weights of feature words in the texts of the training set, which is as follows:

$$\begin{array}{l} w_{i,j}=tf\times idf_{l}‐P_{i}\times pos_{wi}\\ \;=tf\times \log[(\frac{P(o_{i})}{P(n_{i})}\times \frac{P(q_{i})}{P(p_{j})\times \lambda })+1)]\times pos_{wi}\\ \;=\frac{n_{i,j}}{\sum_{k}n_{k,j}}\times \log[(\frac{\frac{df_{i,o}}{N_{o}}}{\frac{df_{i,n}}{N_{n}}+1}\times \frac{\frac{n_{i,j}}{\sum_{N}n_{i,N}}}{\frac{\sum_{k}n_{k,j}}{\sum_{N}(\sum_{k}n_{k,j})}\times \lambda })+1]\times pos_{wi} \end{array}$$ (6)

Since the texts in the test set are used for prediction and the class labels do not exist beforehand in the actual application, the feature word weight calculation of the test set cannot be calculated like that of the training set. Therefore, the weight calculation method of the feature words in the test set is slightly different from that in the training set. In this case, the inverse document frequency is calculated using the traditional *idf* to obtain the other improved formula of TF-IDF-CRF-POS. The formula is as follows:

$$\begin{array}{l} w_{i,j}=tf\times idf‐P_{i}\times pos_{wi}\\ \;=tf\times \log[(\frac{N}{df_{i}}\times \frac{P(q_{i})}{P(p_{j})\times \lambda })+1]\times pos_{wi}\\ \;=\frac{n_{i,j}}{\sum_{k}n_{k,j}}\times \log[(\frac{N}{df_{i}}\times \frac{\frac{n_{i,j}}{\sum_{N}n_{i,N}}}{\frac{\sum_{k}n_{k,j}}{\sum_{N}(\sum_{k}n_{k,j})}\times \lambda })+1]\times pos_{wi} \end{array}$$ (7)

#### Fourth, obtain the algorithmic process of the improvement TF-IDF

Just as mentioned above, the weights of feature words in the training set and test set are calculated slightly differently. Thus, this section proposes the algorithm process of feature term weighting in these two text sets.

(1) The algorithm process for the feature term weight in the training text set:

Step 1: Calculate the ratio of the word frequency *tf* which is the frequency of occurrence *n*_*i*,*j*_ of the word *i* in text *j* to the total number $\sum_{k}n_{k,j}$ of words in that text.

Step 2: Calculate the relative frequency values of P(*q*_*i*_) and P(*p*_*j*_). P(*p*_*i*_) is the ratio of the number of occurrences of the word *i* in the text *j* to the total number of occurrences of the word *i* in the entire text set, while P(*p*_*j*_) is the ratio of the total number of words in that text to the total number of words in the entire text set. It is as shown in Eq 3.

Step 3: Calculate the value of the total relative word frequency P(qi)P(pj)×λ using the results of Step 2, which *λ* is a constant conditioning variable and *λ* = 2.

Step 4: Construct a document classification dictionary based on the annotated classification labels.

Step 5: Use the document classification dictionary to calculate the values of P(*o*_*i*_) and P(*n*_*i*_). P(*o*_*i*_) is the ratio of the number of texts containing the word *i* in the category *o* to the total number of texts in that category *o*, while P(*n*_*i*_) is the ratio of the number of texts containing the word *i* in other categories after excluding the texts of the category *o* to the total number of texts in other categories. It is as shown in Eq 4.

Step 6: Calculate the value of P(oi)P(ni) based on the result of Step 5.

Step 7: Calculate the value of the complete improved inverse document frequency *idf*_*l*_-P_*i*_ by multiplying the results of Step 3 and Step 6, adding 1, and taking the logarithm;

Step 8: Obtain the part-of-speech tagging weighted value *pos*_*wi*_ of each word by substituting the part-of-speech tagging result into Eq 5.

Step 9: The word frequency, the improved reverse document frequency, and the part-of-speech tagging weighted value obtained from Step1, Step 7, and Step 8 are multiplied to obtain the weight value of the word *i* in text *j* of the training set, that is $w_{i,j}=tf\times idf_{l}{‐P}_{i}\times pos_{wi}$, and it is as shown in Eq 6.

Step10: Traverse the text *j*, loop through Step1 to Step9, and calculate the weight value *w*_*k*,*j*_ of each word in the text *j* of the training set.

(2) The algorithm process for the feature term weight in the testing text set:

Step1: Calculate Ndfi, where *N* is the total number of texts in the test text set and *df*_*i*_ is the number of texts containing the word *i* in this text set, and it is as shown in Eq (2);

Step 2: Calculate the value of word frequency *tf*, the value of total relative word frequency P(qi)P(pj)×λ, and the part-of-speech tagging weighted value *pos*_*wi*_ with refering to Step1 to Step3 and Step8 of the feature word weight algorithm of the training text set, then multiply the result of Step1 with the value of total relative word frequency and add 1, and take the logarithm to obtain the value of improved inverse document frequency *idf*-P_*i*_ for word *i* in text *j* of the test set.

Step 3: The word frequency, the improved inverse document frequency, and the POS tagging weighting value obtained from Step 2 are multiplied to obtain the weight value of the word *i* in the text *j* of the test set. That is $w_{i,j}=tf\times idf{‐P}_{i}\times pos_{wi}$, and it is as shown in Eq (7);

Step 4: Traverse the text *j*, loop through the first three steps, and calculate the weight value *w*_*k*,*j*_ of each word in the text *j* of the test set.

In addition, it is worth stating that the method of ’selecting a certain proportion of feature words in order of weight from large to small or selecting feature words whose weight exceed a certain threshold as the feature selection to represent text’, which is commonly used in text classification in many pieces of literature, can reduce the data dimension and training time for the introduction text of tourist attractions, but it does not improve the classification accuracy. As the proportion of selected words approaches the total number of words, the classification accuracy tends to approach that of using all words for classification. Therefore, in this paper, this common method is abandoned to obtain better classification results, and each word is included in the calculation and the feature selection is reflected by the level of their weights.

### 3.4. Generating text sentence vectors

A text is formed by some words connected, whereas the word vectors of description text about tourist attractions are discrete distributions in a high-dimensional space. To obtain continuous and consistent semantic information, it is necessary to combine the word vector and the word weight of each word to generate a high-quality text feature vector, which is often referred to as a text vector or sentence vector. In this paper, we use Word2Vec and TF-IDF to represent the text sentence vector for tourist attraction classification. that is, the feature word weight calculated by the improved TF-IDF model is normalized and multiplied with the word vector of this feature word, and the sentence vector is obtained by summing this result for all feature words with the following equation:

Vdn,vec=∑k[Wk−vec×(wk,j∑kwk,j)] (8)

Where *w*_*k*,*j*_ denotes the weight of the *k* feature word in a text, *W*_*k*−*vec*_ denotes the word vector of the corresponding *k* feature word, and the division of *w*_*k*,*j*_ by $\sum_{k}w_{k,j}$ is done so that the weights are in the (0,1) interval for normalization purposes.

The algorithm flow for generating text sentence vectors is as follows:

Step1: Divide the experimental text set into training set samples and test set samples;

Step 2: Use the improved TF-IDF-CRF-POS①, namely Formula (6), to calculate the weight value *w*_*k*,*j*_ of each feature word in the text *j* of the training set according to the algorithmic processes of feature word weight of the training text set.

Step 3: Normalize the weight value obtained from Step 2 by dividing it by the sum of the weights $\sum_{k}w_{k,j}$ of all the terms in the document.

Step 4: Multiply the normalized weights obtained from Step 3 by their corresponding word vectors *W*_*k*−*vec*_ from the Word2Vec model trained to obtain the weighted word vectors.

Step5: Sum the weighted word vectors of all words in the text *j* to get the text representation vector $V_{d_{n},vec}$ of this text *j* (according to Eq 8), because each line is a document in the calculation, it can also be called the sentence vector, then to get the sentence vector of 1*m dimension.

Step 6: Iterate over each of the *n* texts in the text set to obtain the n*m dimensional text vector matrix corresponding to the text set.

Step 7: Use the improved TF-IDF-CRF-POS②, namely Formula (7), to obtain the sentence vectors of the test text set according to Step2 to Step6.

### 3.5. Classifiers and evaluation metrics

To verify the effectiveness, superiority, and feasibility of the improved model, this paper compares it experimentally with three commonly used text representation methods, such as TF-IDF+Word2Vec, average weight+Word2Vec, and Doc2vec. Different text representation methods need to be paired with different classification models to achieve optimal results. Therefore, in this paper, seven commonly used and well-performing ML classification algorithms, including Decision Tree (DT), Support Vector Machine (SVM), Logistic Regression (LR), Naive Bayes (NB), Multilayer Perceptron (MLP), Random Forest (RF), and K-Nearest Neighbor (KNN), are selected to classify the dataset and do comparative analysis.

Classification models are often evaluated using indicators such as Precision, Recall, F1-measure, and Accuracy. The first three indicators are classification indicators, which are mainly for the evaluation of a certain category. In multi-classification problems, these three classification indicators are usually subjected to Micro Average, Macro Average, or Weighted Average to evaluate the overall classification effectiveness of the algorithm. All evaluation metrics are calculated based on the binary contingency table (as in Table 1), where TP represents the number of texts that actually belong to the category and are correctly classified, FP represents the number of texts that actually do not belong to the category but are incorrectly classified as this category, FN represents the number of texts that actually belong to the category but are incorrectly classified as other categories, and TN represents the number of texts that actually do not belong to the category and are correctly classified as other categories. The calculation methods of all evaluation indicators are shown in Tables 2 and 3 [39]. The weighted average is the average indicator value obtained by assigning different weights to each category according to the proportion of the true distribution of different categories, and then multiplying the corresponding indicators of each category and adding them together. Because the dataset used in this paper is imbalanced (as analyzed in the following text), the paper adopts the weighted average metric to evaluate the model. It considers the imbalance of categories and can present the classification results of imbalanced datasets more objectively. At the same time, the evaluation indexes of the Macro-average and Accuracy of the overall model are supplemented to comprehensively analyze and evaluate of the effect of the classification algorithm. Among them, the four index values of Accuracy of the overall model, Precision, Recall, and F1-measure of the micro-average are equal.

**Table 1:** Binary contingency table.

	Actually in this category	Not actually in this category
**Judged to be in this category**	*TP*	*FP*
**Judged to not be in this category**	*FN*	*TN*

**Table 2:** Evaluation indicators -1 for classification results.

Evaluation Indicators	Precision *P*	Recall *R*	F-measure *F*_*β*_	F1-measure (*β* = 1)	Accuracy
**Calculation formula**	$\frac{TP}{TP+FP}$	$\frac{TP}{TP+FN}$	$\frac{(\beta^{2}+1)\;PR}{\beta^{2}P+R}$	$\frac{2PR}{P+R}$	$\frac{TP+TN}{TP+FP+FN+TN}$

**Table 3:** Evaluation indicators -2 for classification results.

**Evaluation Indicators**	**Macro Average**			**Micro average**		
	**Precision *macro-P***	**Recall *macro-R***	**F1 *macro-F1***	**Precision *micro-P***	**Recall *micro-R***	**F1 *micro-F1***
**Calculation formula**	$\frac{1}{n}\sum_{i=1}^{n}P_{i}$	$\frac{1}{n}\sum_{i=1}^{n}R_{i}$	$\frac{2\times MacroP\times MacroR}{MacroP+MacroR}$	$\frac{\sum_{i=1}^{n}TP_{i}}{\sum_{i=1}^{n}TP_{i}+\sum_{i=1}^{n}FP_{i}}$	$\frac{\sum_{i=1}^{n}TP_{i}}{\sum_{i=1}^{n}TP_{i}+\sum_{i=1}^{n}FN_{i}}$	$\frac{2\times MicroP\times MicroR}{MicroP+MicroR}$
**Evaluation Indicators**	**Weighted average**					
	**Precision *weighted-P***		**Recall *weighted-R***	**F1 *weighted-F1***		
**Calculation formula**	$\frac{1}{n}\sum_{i=1}^{n}\left(P_{i}\times W_{i}\right)$		$\frac{1}{n}\sum_{i=1}^{n}\left(R_{i}\times W_{i}\right)$	$\frac{2\times WeightedP\times WeightedR}{WeightedP+WeightedR}$		

## 4. Experimental procedure and results analysis

### 4.1. Experimental environment

The computer configuration used for the experiments in this paper is 11thGenIntel® CoreTMi5-5200UCPU@2.50GHz processor, 16GB RAM, Windows 11 Home Edition 64-bit OS, 512GB SSD, and the development tool is PyCharm2021, using Python programming language, version Python 3.6, and using the toolkits such as Jieba, Pickle, Sklearn, Numpy, Pandas, and Gensim, etc. The same computer was used for all experiments.

### 4.2. Experimental data

After collecting and organizing the list of national A-level tourist attractions, this paper obtained a final list of 13,654 attractions in 31 provincial-level administrative regions of China (excluding Hong Kong, Macao, and Taiwan). Seven provinces or cities (Shanghai, Hubei, Hunan, Sichuan, Yunnan, Guizhou, and Guangdong) were randomly selected as research samples, and the introduction texts of A-level tourist attractions in these places were collected by Python web crawler technology and pre-processed to obtain 3,498 description texts of tourist attractions as the experimental dataset, with a total vocabulary of 203,187. Then, 80% of the texts in the dataset were selected as the training set (2,798 items), and the remaining 20% were selected as the test set (700 items), and every tourist attraction was manually labeled with its category to verify the effectiveness of the improved model. In China, tourist attractions are commonly classified into dichotomous, trichotomous, and quintuple categories. This paper mainly refers to the quintuple classification method [40], which divides tourist attractions into five categories based on the main type of tourism resources: nature, culture, composite, theme park, and society categories. Additionally, due to the emerging and popular trend of resort tourist attractions, this paper includes them as a sixth category in the tourist attractions classification.

**Fig 4:**
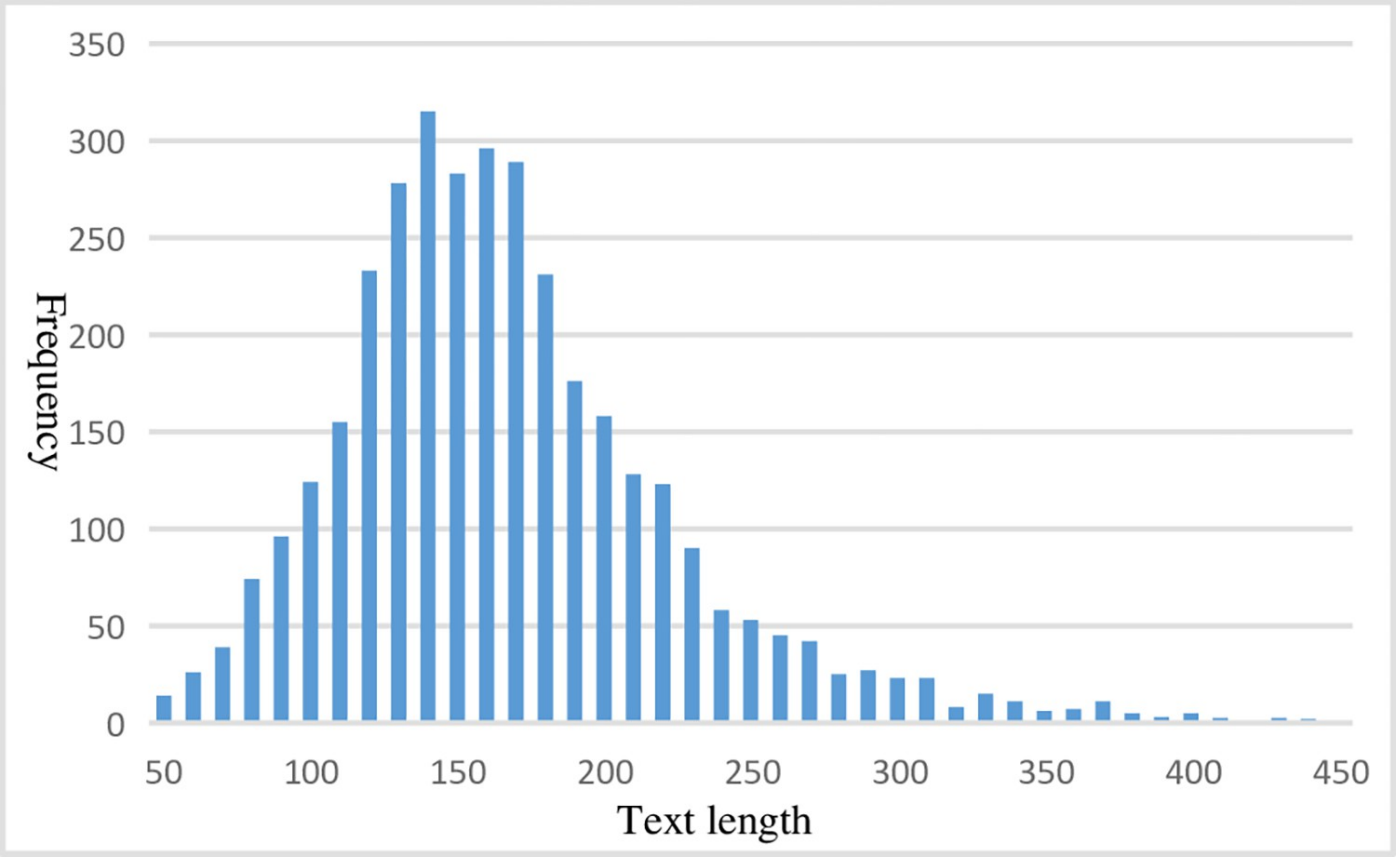
Frequency of occurrence of different text lengths.

In this paper, Fig 4 shows the data visualization analysis of the 3498 experimental texts. It can be observed that the majority of the texts are under 300 characters (97.20%). According to the criterion of "short texts in Chinese generally refer to texts less than 140 Chinese characters", a significant portion (38.71%) of texts in the text set belong to short texts, while the length of the remaining texts is between 140 and 440 Chinese characters, accounting for 61.29%. Therefore, the text set used in this paper is a complex text set, comprising two types of texts: short and mediumlength. Statistically, the average length of each experimental text is 162 characters, with the shortest text being 28 characters and the longest being 440 characters. Furthermore, the distribution of different categories of experimental texts is imbalanced (as shown in Table 4), and the number of valid texts among them varies significantly. In particular, the composite category has the least number of samples, while the cultural category has the greatest number of samples, with a difference of nearly 5 times between the two. This indicates that the experimental dataset in this paper is imbalanced or skewed, and text classification of this type of dataset is more challenging than that of a balanced dataset.

**Table 4:** Distribution of experimental dataset categories.

Category number	Category	Number of valid texts	Category number	Category	Number of valid texts
0	Nature	984	3	Theme park	325
1	Culture	1039	4	Society	660
2	Composite	178	5	Resort	312

### 4.3. Hyperparameter setting

The experimental parameters in this paper mainly involve the parameters of word and sentence vector training models and classification models. The parameter tuning results were obtained by referring to the parameter settings of related literature, combining Grid search and experimental verification, as shown in Tables 5–7. For example, for the dimension setting of word vector, this paper experimentally tried different dimensionality settings such as 50, 100, 256, 300, and 600. It was found that the F1-measure with higher dimensions was not higher than that with 100 dimensions. To improve the training speed and the computational speed of the following steps, the dimensionality of the word vector is set to 100 in this paper. The important parameters of the seven classifiers are shown in Table 6, and the remaining parameters for these classifiers adopt their default values. The BERT model in this paper is based on the Chinese-bert-wwm pre-training model released by Joint Laboratory of HIT and iFLYTEK Research, and its hyperparameter settings are detailed in Table 7.

**Table 5:** Hyperparameter settings for Word2Vec and Doc2vec.

Parameter Name	Parameter description	Setting
size	Dimension of word vector	100
window	Window size of the context	5
min-count	Minimum threshold of word occurrence	1
sg	Training algorithm for Word2Vec (1 for using Skip-gram model)	1
workers	Number of parallelism in training	10
iter	Number of iterations	20
vector_size	Sentence vector dimension of Doc2vec	100
dm	Training algorithm for Doc2vec (0 for using PV-DBOW model)	0

**Table 6:** Hyperparameter settings of classification model.

Model	Hyperparameter settings
DT	criterion = entropy, max_depth = 7, min_samples_leaf = 7, min_impurity_decrease = 0.0032, random_state = 30
SVM	gamma = 0.01, C = 1, random_state = 30
LR	solver = saga, C = 0.2, random_state = 30
NB	No parameters, and no need to adjust parameters
MLP	solver = adam, alpha = 1.41, activation = relu, random_state = 30
RF	criterion = entropy, max_depth = 12, min_impurity_decrease = 0.0004, max_features = sqrt, random_state = 30
KNN	n_neighbors = 12, n_jobs = 5

**Table 7:** Hyperparameter settings of BERT model.

Hyperparameter	Parameter description	Setting
dropout	Random discard rate	0.1
lr	Learning rate	2e-5
eps	Numerical stability in the Adam optimizer	1e-8
weight_decay	Weight decay or regularization Parameters	1e-3
batch_size	Lot size	10
epoch	Number of training iterations	5
optimizer	Optimizer	Adam
max_length	Maximum text length	442
add_special_tokens	Whether to add special placeholders	True
pad_to_max_length	Whether the text is filled to the maximum length	True
bert_embdding	Dimension of word vector	768

### 4.4. Comparative analysis of experimental results

The comparison experiment in this paper consists of four parts. The first part is the comparison experiment of the improvement process, which illustrates the improvement effect of each step of this method in this paper. The second part is the comparison of model effect, where the proposed method TF-IDF-CRF-POS+Word2Vec is compared with the traditional TF-IDF+Word2Vec, average weight+Word2Vec, and Doc2vec from the aspects of overall effect and specific category performance, to illustrate the effectiveness and superiority of the proposed method. The third part is stability comparison——comparing the model effects of different-sized text sets to demonstrate the robustness of the proposed method. The fourth part is the comparison of prediction application conclusions, which analyzes the differences between the predicted value and the real value after using this proposed method to predict the text classification labels, demonstrating the feasibility of the application of the improved model. When evaluating the quality of the model, if the conclusions of the overall accuracy, the macro-average F1-measure, and the weighted average F1-measure are inconsistent, the weighted average F1-measure will be taken as the final evaluation standard.

#### 4.4.1. Experimental comparison of improved processes

As mentioned above, the TF-IDF-CRF-POS algorithm in this paper is improved by gradually introducing factors such as total relative word frequency, category differentiation, and part of speech in three steps based on the traditional TF-IDF model. This part of the experiment adopts the method of controlling variables to compare the improvement effect of the classification performance in the entire test set and in each category as the above three steps are progressively advanced in the same Word2Vec training model and the MLP classifier (as shown in Tables 8 and 9). It can be seen from Table 8 that the overall accuracy, macro-F1, and weighted-F1 have steadily improved in each step compared to the previous step, among which the weighted F1 has been improved by -0.18% (Explanatory note: Although the weighted-F1 value here is slightly lower than that in the previous step, it does not mean that the improvement of total relative word frequency is reversed. That is because some indicators, such as macro-R, weighted-R, and some evaluation indicators of specific categories (e.g., theme park category and nature category), have improved. In addition, the experimental results show that the weighted-F1 value can only reach 81.55% if the improvement of total relative word frequency is removed. This indicates that the three improvement steps are complementary and indispensable), 3.20% and 0.46%, respectively, with a total increase of 3.48% in the three steps. Compared with the corresponding value of the traditional TF-IDF model, every evaluation index of the final improved model shows a different degree of improvement, ranging from 1.51% to 7.58%. This indicates that the method in this paper has achieved the purpose of comprehensively improving the classification effect of description text in terms of accuracy, precision, and recall. Therefore, the improvement is effective. Among them, although the macro-precision has decreased compared to the previous step when category differentiation is introduced, the macro-recall has increased. The reason for this is that, on the one hand, there is a negative correlation between precision and recall, and on the other hand, the addition of category differentiation improves the category recognition rate and thus improves its recall. Especially for the categories with low recall, the improvement effect is more significant. For example, for the composite category with the lowest recall (as shown in Table 9), its recall immediately increased by 35.42% after the category improvement compared to the initial state. In general, after three steps of improvement, the model can significantly improve the recall for categories with low recall (e.g., composite category, theme park category, and resort category), while for categories with high recall (e.g., nature category and culture category), the recall is slightly reduced in exchange for a significant increase in precision, thereby ultimately improving the F1-measure of the category.

**Table 8:** Classification performance of the entire test set in the MLP classifier during the improved processes.

Serial number	Improved process	Acc	Macro avg			Weighted avg		
			P	R	F1	P	R	F1
①	Traditional TF-IDF+Word2Vec	86.43%	85.46%	76.77%	78.60%	86.72%	86.43%	85.47%
②	Introduce the total relative word frequency based on ①	86.43%	86.08%	76.72%	78.09%	87.05%	86.43%	85.29%
③	Improve category differentiation based on ②	88.57%	85.57%	83.30%	**84.19%**	88.68%	88.57%	**88.49%**
④	Introduce the distinctions of word nature based on ③	89.00%	86.97%	84.35%	**85.37%**	89.18%	89.00%	**88.95%**
④-① Difference of the total effect of improvement		2.57%	**1.51%**	**7.58%**	6.77%	2.46%	2.57%	3.48%

**Table 9:** Classification performance of each category of the test set in MLP during the improved processes.

Serial number	Improved Process		Theme park	Culture	Composite	Resort	Society	Nature
①	Traditional TF-IDF +Word2Vec	P	74.32%	91.16%	94.12%	83.78%	82.41%	86.98%
		R	76.39%	94.19%	33.33%	75.61%	83.18%	97.91%
		F1	75.34%	92.65%	49.23%	79.49%	82.79%	92.12%
②	Introduce the total relative word frequency based on ①	P	75.00%	90.80%	100.00%	80.00%	83.65%	87.04%
		R	79.17%	94.19%	29.17%	78.05%	81.31%	98.43%
		F1	77.03%	92.46%	45.16%	79.01%	82.46%	92.38%
③	Improve category differentiation based on ②	P	74.39%	94.89%	82.50%	86.11%	85.29%	90.24%
		R	84.72%	92.53%	68.75%	75.61%	81.31%	96.86%
		F1	79.22%	93.70%	**75.00%**	80.52%	83.25%	93.43%
④	Introduce the distinctions of word nature based on ③	P	74.39%	94.54%	89.47%	89.19%	82.69%	91.54%
		R	84.72%	93.36%	70.83%	80.49%	80.37%	96.34%
		F1	79.22%	93.95%	**79.07%**	84.62%	81.52%	93.88%
④-①Difference of improvement effect		P	0.07%	3.38%	-4.65%	5.41%	0.28%	4.56%
		R	**8.33%**	-0.83%	**37.50%**	**4.88%**	-2.81%	-1.57%
		F1	3.88%	1.30%	29.84%	5.13%	-1.27%	1.76%

#### 4.4.2. Model effect comparison

*(1) Overall assessment results*. Different text representation methods need to be combined with different classifiers to achieve optimal performance. The classification performance of various combinations on the entire test set is presented in Table 10, allowing for a comparison between the improved TF-IDF-CRF-POS+Word2Vec model in this paper and the traditional TF-IDF+Word2Vec model, average weight+Word2Vec model, and Doc2vec model, in terms of their superiority and inferiority. The advantages of the improved method are summarized as follows:

**Table 10:** Classification performance of different combinations of text representation method & classifier.

Classifier Text represent- ation method			DT		SVM	LR	RF	MLP	NB	KNN
Traditional TF-IDF +Word2Vec	accuracy		***72*.*14%***		85.86%	86.29%	83.86%	**86.43%**	79.43%	81.43%
	macro	P	61.28%		86.24%	86.52%	85.51%	85.46%	76.10%	78.96%
		R	59.26%		75.42%	76.96%	70.10%	76.77%	76.93%	67.79%
		F1	***59*.*59%***		77.24%	**78.88%**	71.31%	78.60%	74.48%	69.37%
	weighted	P	71.04%		86.42%	86.83%	84.79%	86.72%	84.04%	81.04%
		R	72.14%		85.86%	86.29%	83.86%	86.43%	79.43%	81.43%
		F1	***71*.*25%***		84.55%	85.30%	81.37%	**85.47%**	80.59%	79.40%
TF-IDF-CRF-POS +Word2Vec	accuracy		76.43%		86.57%	87.86%	84.43%	**89.00%**	***63*.*43%***	85.00%
	macro	P	68.54%		84.31%	84.70%	81.92%	86.97%	72.84%	81.48%
		R	65.34%		79.19%	81.69%	71.47%	84.35%	65.27%	76.56%
		F1	65.86%		81.07%	82.98%	73.76%	**85.37%**	***61*.*17%***	78.43%
	weighted	P	75.20%		86.72%	87.80%	83.99%	89.18%	82.66%	84.60%
		R	76.43%		86.57%	87.86%	84.43%	89.00%	63.43%	85.00%
		F1	75.31%		86.33%	87.72%	82.84%	**88.95%**	***67*.*90%***	84.49%
Average weight + Word2Vec	accuracy		***71*.*14%***		84.43%	**85.71%**	83.29%	85.14%	79.57%	81.71%
	macro	P	55.69%		82.95%	85.86%	87.43%	84.35%	74.73%	77.62%
		R	54.76%		74.13%	77.22%	68.09%	76.71%	75.10%	68.62%
		F1	***54*.*19%***		75.89%	**79.27%**	70.28%	78.71%	73.81%	70.43%
	weighted	P	67.16%		84.22%	86.39%	84.67%	85.34%	82.26%	80.91%
		R	71.14%		84.43%	85.71%	83.29%	85.14%	79.57%	81.71%
		F1	***68*.*50%***		83.10%	**85.02%**	80.56%	84.41%	80.26%	79.97%
Doc2vec	accuracy		***68*.*86%***		80.57%	**82.57%**	78.00%	80.29%	77.29%	77.00%
	macro	P	54.58%		74.68%	80.80%	71.87%	75.68%	71.48%	69.72%
		R	54.35%		68.16%	70.92%	62.18%	68.26%	72.85%	63.09%
		F1	***53*.*88%***		67.61%	**72.50%**	62.62%	68.84%	71.50%	62.50%
	weighted	P	66.20%		79.20%	82.36%	75.89%	79.80%	79.43%	75.01%
		R	68.86%		80.57%	82.57%	78.00%	80.29%	77.29%	77.00%
		F1	***67*.*23%***		78.16%	**81.00%**	74.90%	78.65%	77.86%	74.23%
BERT	accuracy	Mac-P		Mac-R		Mac-F1	Wei-P	Wei-R	Wei-F1	
	**86.71%**	83.34%		77.54%		**79.82%**	86.23%	86.71%	**86.05%**	

Note: Only bold indicates the highest value in the classifier, italic bold indicates the lowest value in the classifier.

#### Firstly, the macro average values are the lowest among the three kinds of average values due to the influence of rare categories, but the improved method can narrow the gap between the three kinds of average values

**Fig 5:**
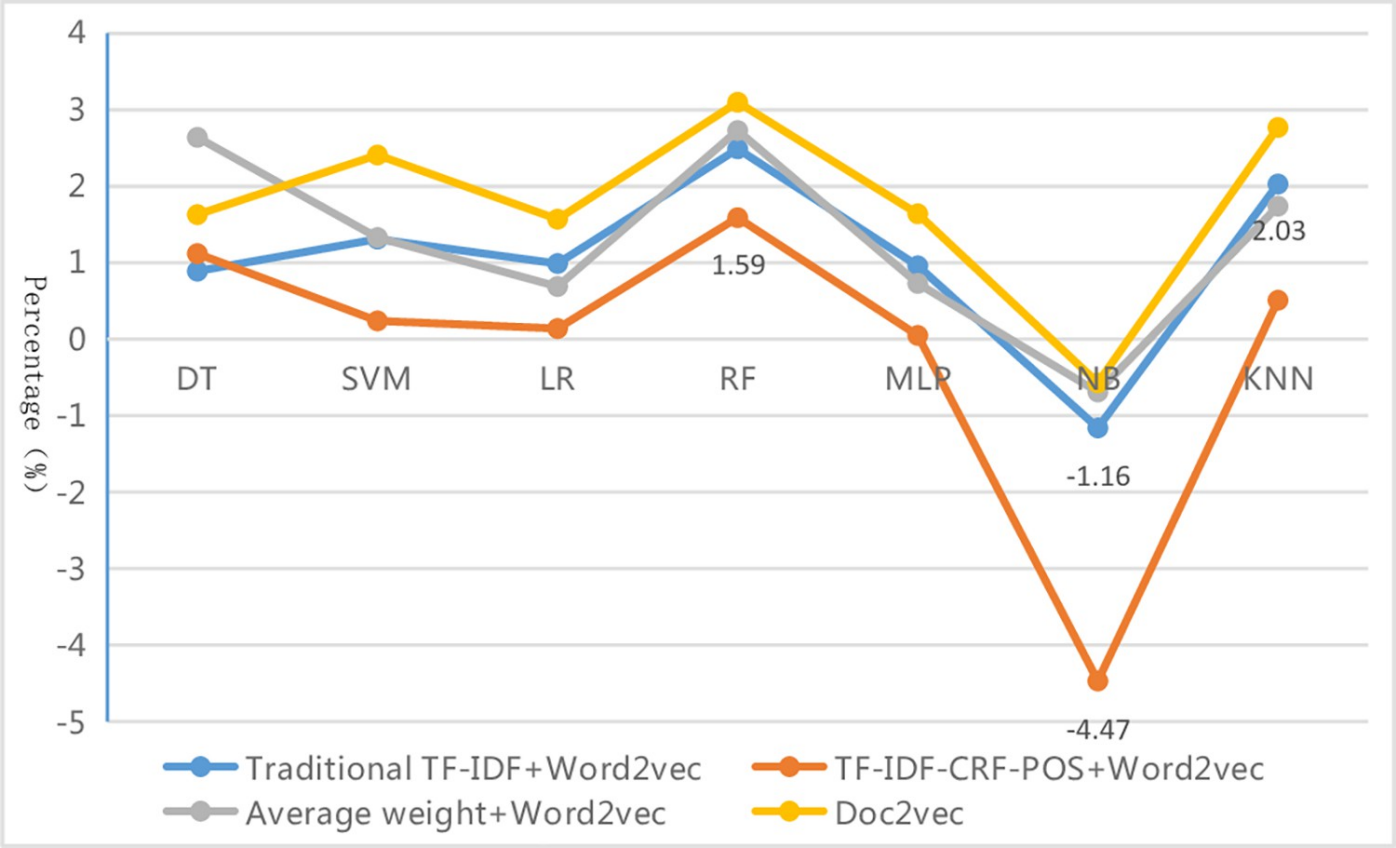
Difference line graph of "micro-F1 minus weighted-F1".

**Fig 6:**
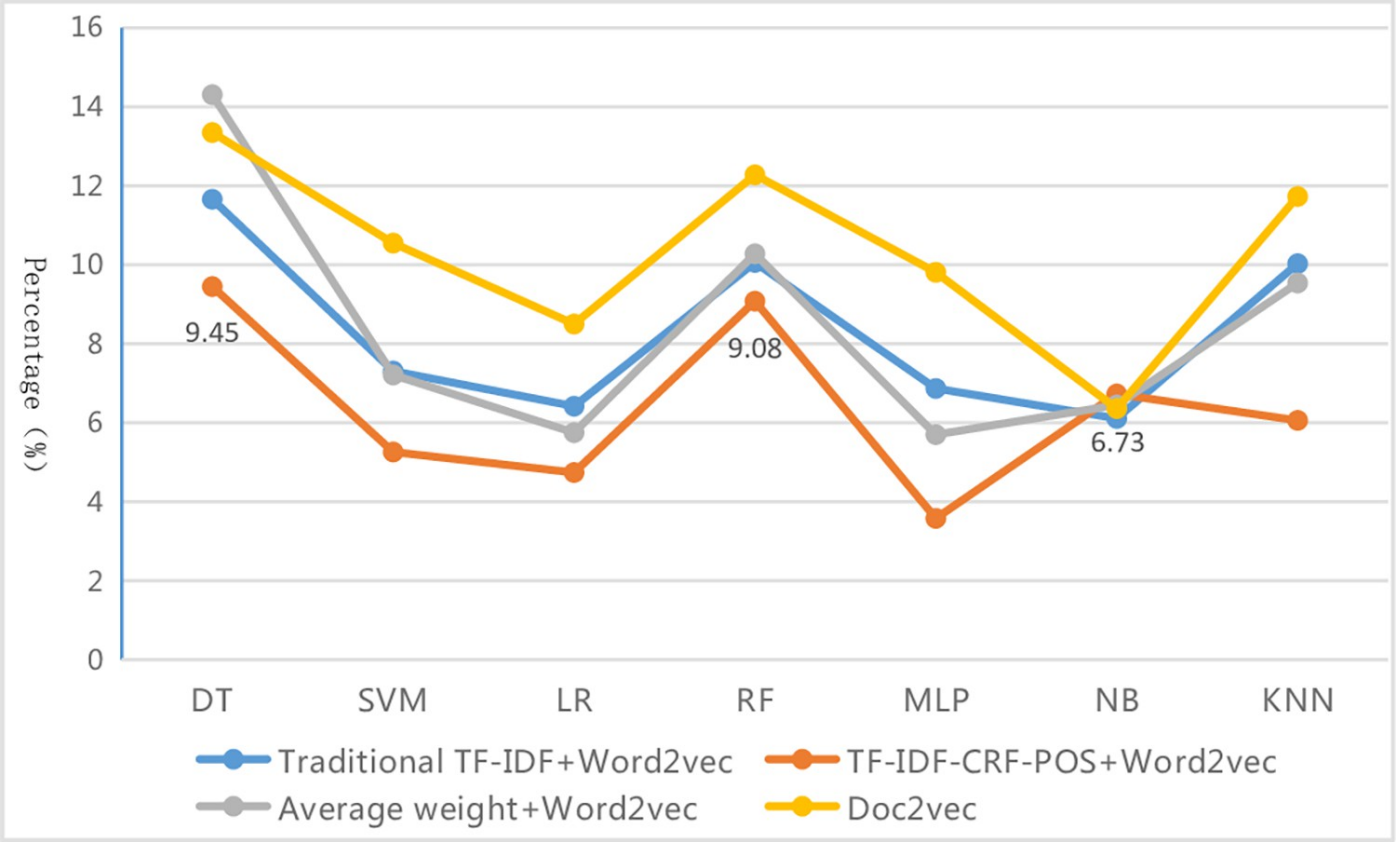
Difference line graph of "weighted-F1 minus macro-F1".

From Table 10, it can be seen that each evaluation index value of different text representations under different classifiers roughly shows the characteristics of micro average > weighted average > macro average. That is, the precision, recall, and F1-measure of the macro average are the lowest among the three kinds of average values. This is mainly because the macro average values are affected by rare categories, which are under-learned due to their small sample size, resulting in lower precision, recall, and F1-measure for those categories, thus dragging down the corresponding metric values of the macro average. Among these methods, each evaluation metric value of the micro average is the highest, while that of the weighted average is the second, and the gap between them is small. This is because metric values of the micro average are equal to the accuracy of the whole sample, making them more susceptible to the influence of common categories, just like the weighted average in the imbalanced dataset. Due to the large sample size and sufficient learning, the common categories have high precision, recall, and F1-measure, and this elevates the corresponding metric values of the micro average and weighted average. The difference values of "micro-F1 (accuracy) minus weighted-F1" and "weighted-F1 minus macro-F1" under different classifiers are further calculated and shown in Figs 5 and 6. The results show that under various classifiers, the two kinds of difference values of the proposed method in this paper are the smallest overall, although this is not the case in individual instances within the four methods. This indicates that the improved method has reduced the differences in classification performance among various categories in the imbalanced dataset and has made the gap between the three types of average index values smaller. Therefore, the evaluation results are more consistent and accurate, further proving the superiority and robustness of this proposed method from different aspects. By examining the specific data, it is found that the reduction in the gap is mainly due to a significant increase in the various assessment indicators values of rare categories, as will be presented in the analysis of specific categories below.

#### Secondly, different text representation methods are suitable for different classifiers, but the improved method performs better under different classifiers

**Fig 7:**
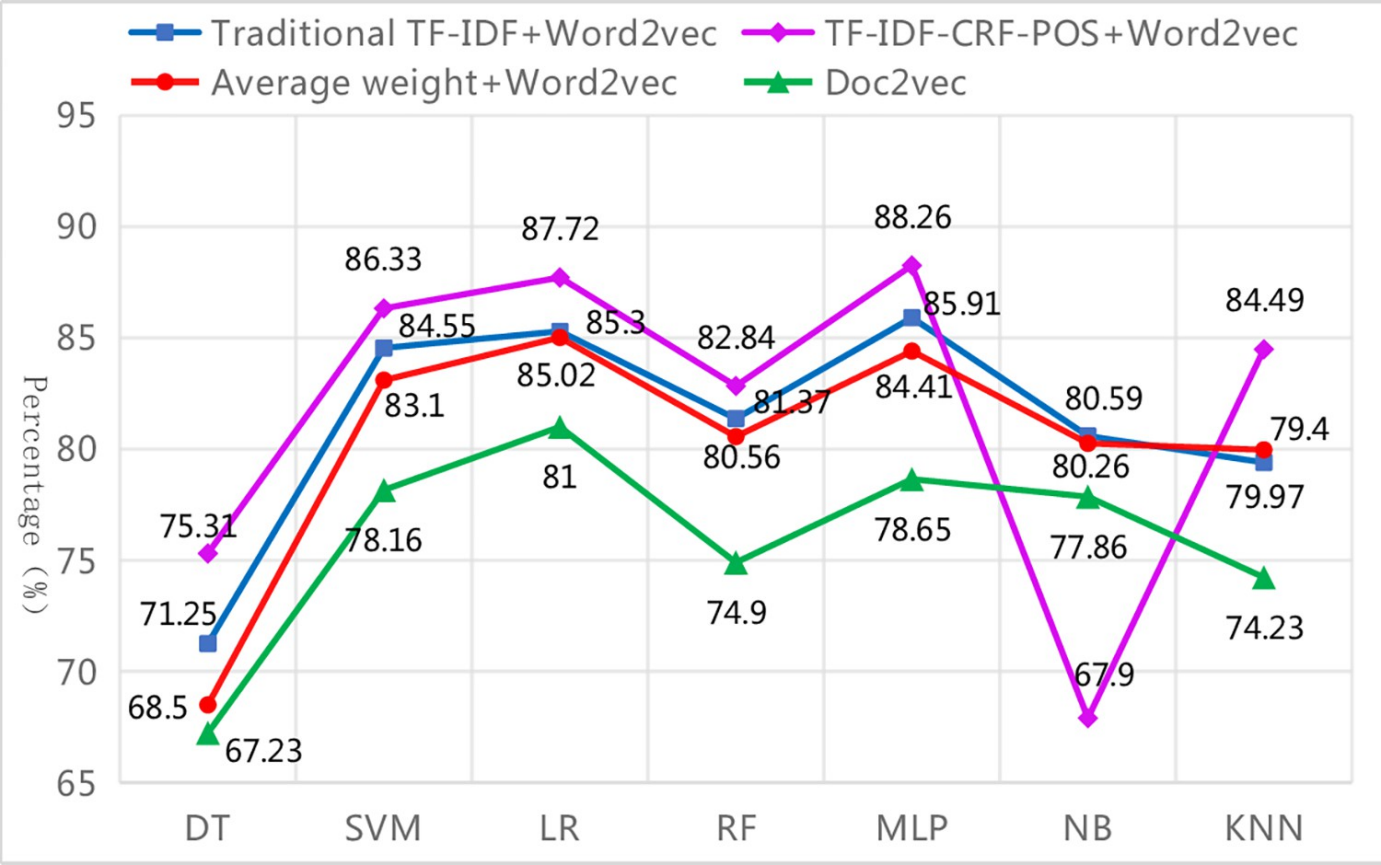
Weighted-F1 values for different combinations of text representations & classifiers.

As seen in Table 10, the top three classifiers with the best performance for the text representation methods of traditional TF-IDF+Word2Vec and TF-IDF-CRF-POS+Word2Vec are MLP, LR and SVM, respectively. Similarly, for the text representation methods of average weight+Word2Vec and Doc2vec, the top three suitable classifiers are LR, MLP, and SVM respectively, which are slightly different from the previous two text representation methods. The performance of the DT classifier is either the worst or the second worst for all four text representation methods, indicating that the DT classifier is not suitable for text classification of imbalanced datasets. The main reason is that when certain categories dominate the text classification, the DT classifier creates a biased tree, leading to inaccurate classification results and reduced classifier performance. From Fig 7, it can be observed that among the six other classifiers excluding NB, TF-IDF-CRF-POS+Word2Vec exhibits better classification performance than other text representation methods, followed by traditional TF-IDF+Word2Vec and average weight+Word2Vec, while Doc2vec performs the worst. Two conclusions can be drawn from this. First, the proposed method is not suitable for text classification when combined with DT or NB classifiers, especially NB, as well as NB also performs poorly with other methods. This is because the NB classifier assumes that the feature items are independent of each other, but in reality, the feature items obtained by the four text representation methods are not independent due to considering the contextual semantic and syntactic relations of the feature words. This contradicts the assumption and results in poor classification effectiveness. Second, Doc2vec demonstrates the worst performance, indicating that although Doc2vec considers the overall semantics of paragraph text, it might be useful for calculating text similarity, but it disregards the importance of words and the classification information derived from word representation in text classification tasks. As a result, its application effect in the classification system is poor.

#### Thirdly, in all the optimal classification combination models, the improved method demonstrates the best classification performance

**Fig 8:**
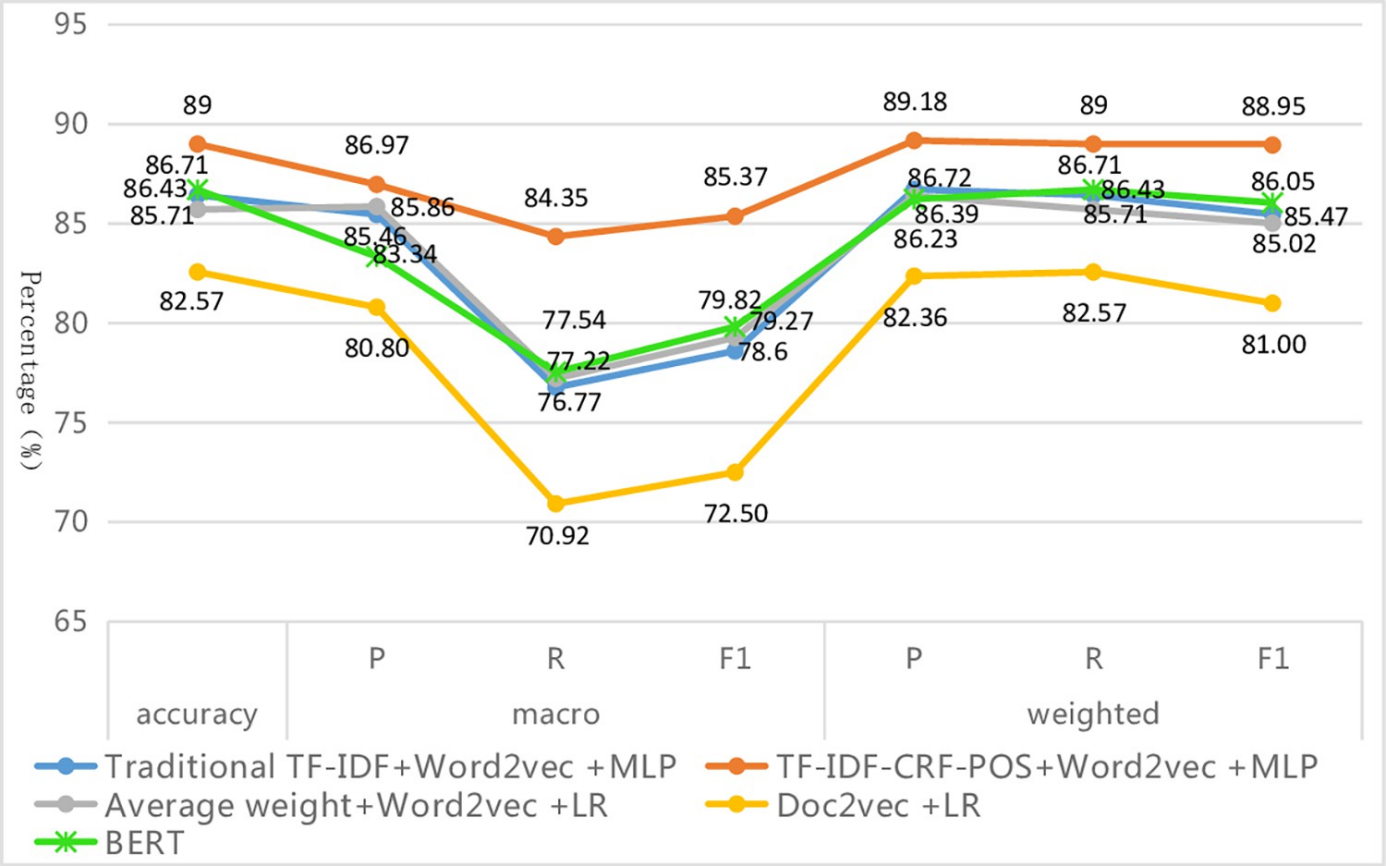
Values of each evaluation index under the optimal classification combination model.

The evaluation metric values obtained by the optimal combination of different text representation methods and classifiers are shown in Fig 8. At this point, the overall comparison of various algorithms is as follows (‘>‘ means ‘superior’): TF-IDF-CRF-POS+Word2Vec+MLP algorithm > traditional TF-IDF+Word2Vec+MLP algorithm > average weight + Word2Vec+LR algorithm > Doc2vec+LR algorithm. In other words, the text classification combination algorithm proposed in this paper achieves the highest values on all seven evaluation metrics, and its precision, recall, and F1-measure of weighted average can reach approximately 89%. These values are 2.46%, 2.57%, and 3.48% higher respectively than those of the traditional TF-IDF+Word2Vec+MLP algorithm, which demonstrates the best performance among the other three algorithms. Furthermore, they are 6.82%, 6.43%, and 7.95% higher respectively than those of the Doc2vec+ LR algorithm, which exhibits the worst performance. The improved method shows the greatest improvement in macro-R among the seven evaluation indicators, with respective increases of 7.58%, 7.13%, and 13.43% compared with the other three methods. This indicates that the improved method in this paper greatly enhances the recall of rare categories and thus improves the overall macro-R value.

Furthermore, this section also conducts a comparison experiment with the BERT classification model, which is currently widely acclaimed and performs excellently in the industry. It was found that although the fine-tuned BERT model’s performance surpasses that of the other three methods, it was still inferior to the TF-IDF-CRF-POS+Word2Vec +MLP algorithm proposed in this paper, with Acc, marco-F1, and mirco-F1 values being 2.29%, 5.55%, and 2.90% lower, respectively. This may be due to the lack of specialized corpora such as tourist attraction description texts in the BERT model’s pre-training, and there are significant differences between the domain-specific data of tourist attraction description texts and the pre-training data of the BERT model in terms of language style, terminology or expression habits, vocabulary usage frequency, and sentence structure complexity. Although fine-tuning has been applied, the model still struggles to adapt to these changes, resulting in poor transfer learning performance. The large difference in the marco-F1 value is because the BERT model’s performance on rare categories is far inferior to that of the proposed method. For example, the F1 value of the BERT model’s composite category is only 50%, which is 29.07% lower than that of the proposed method. In addition, because the BERT model contains a large number of parameters, it is relatively complex, which can lead to excessive computational and memory overheads. Even if only fine-tuning training is performed on BERT, it still takes a long time. In contrast, the structure of the proposed method is more lightweight and has superior execution efficiency. Specifically, under the same experimental environment and hardware equipment, the total runtime of all steps in the proposed method is only 896s, whereas the fine-tuning training and classification prediction of BERT requires 5394s. Notably, as the dataset size increases, the advantages of the proposed method become even more pronounced. This indicates that although the latest NLP models such as BERT perform well on a wide range of tasks, more professional models or domain-specific pre-training may be required for specific domain tasks. At the same time, specific tasks need to be considered comprehensively in light of actual situations, and classic models cannot be completely abandoned. Improved classic models may achieve better performance and efficiency, especially when applied in specialized fields such as tourist attractions.

**Fig 9:**
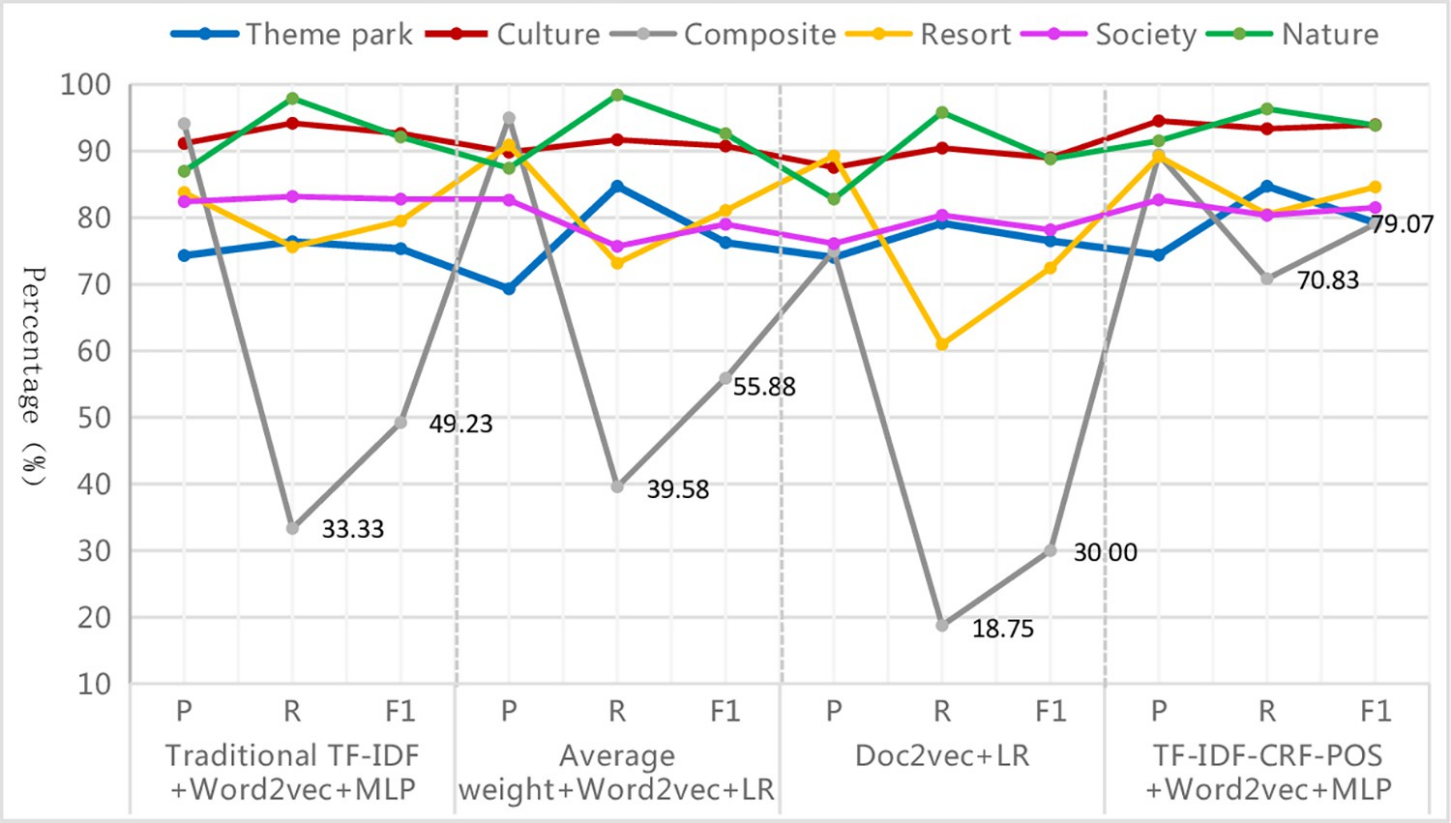
Classification results of each category in the optimal combination of different text representations & classifiers.

**Fig 10:**
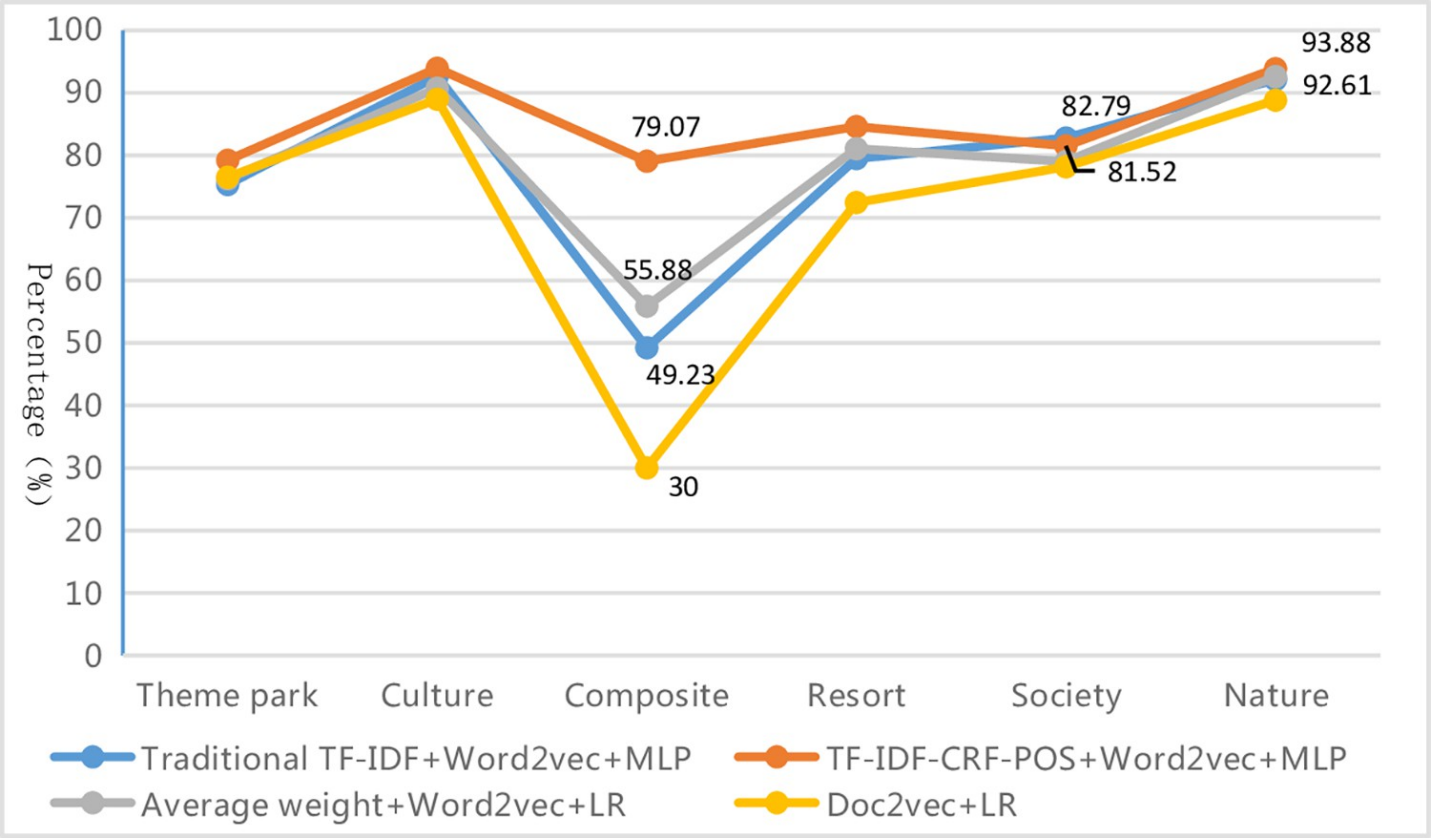
F1-measure of each category in the optimal combination of different text representations & classifiers.

*(2) Evaluation results of specific categories*. In these optimal classification combination algorithms, the classification effect of specific categories is shown in Fig 9. It can be seen that the improved method in this paper has improved the precision, recall, and F1-measure values of the rare categories with poor classification effects in different degrees, such as the theme park category (Support 72), resort category (Support 41) and composite category (Support 48), especially for the worst-performance composite category, which has the most obvious effect on the improvement of the recall and F1-measure values. The recall and F1-measure of the improved model are respectively 37.50% and 29.84% higher than those of the traditional TF-IDF+Word2Vec+MLP algorithm, 31.25% and 23.19% higher than those of the average weight+Word2Vec+LR algorithm, and even 50% higher than those of the worst-performing Doc2vec+LR algorithm. This indicates that the improved method can effectively identify rare categories, learn enough information, and extract sufficient features to obtain better classification results even with a small sample size, making it more robust than the other three methods. Additionally, the overall fluctuation range of the evaluation index values curve for all categories of the improved method is the smallest, and its F1-measure for all categories is the highest except for " F1-measure of the social category, which is slightly lower than that of the traditional TF-IDF + Word2Vec + MLP algorithm by about 1% and ranks the second" (as shown in Fig 10). This indicates that the improved method demonstrates the best and most stable classification performance for each category on the whole.

#### 4.4.3. Stability comparison

**Fig 11:**
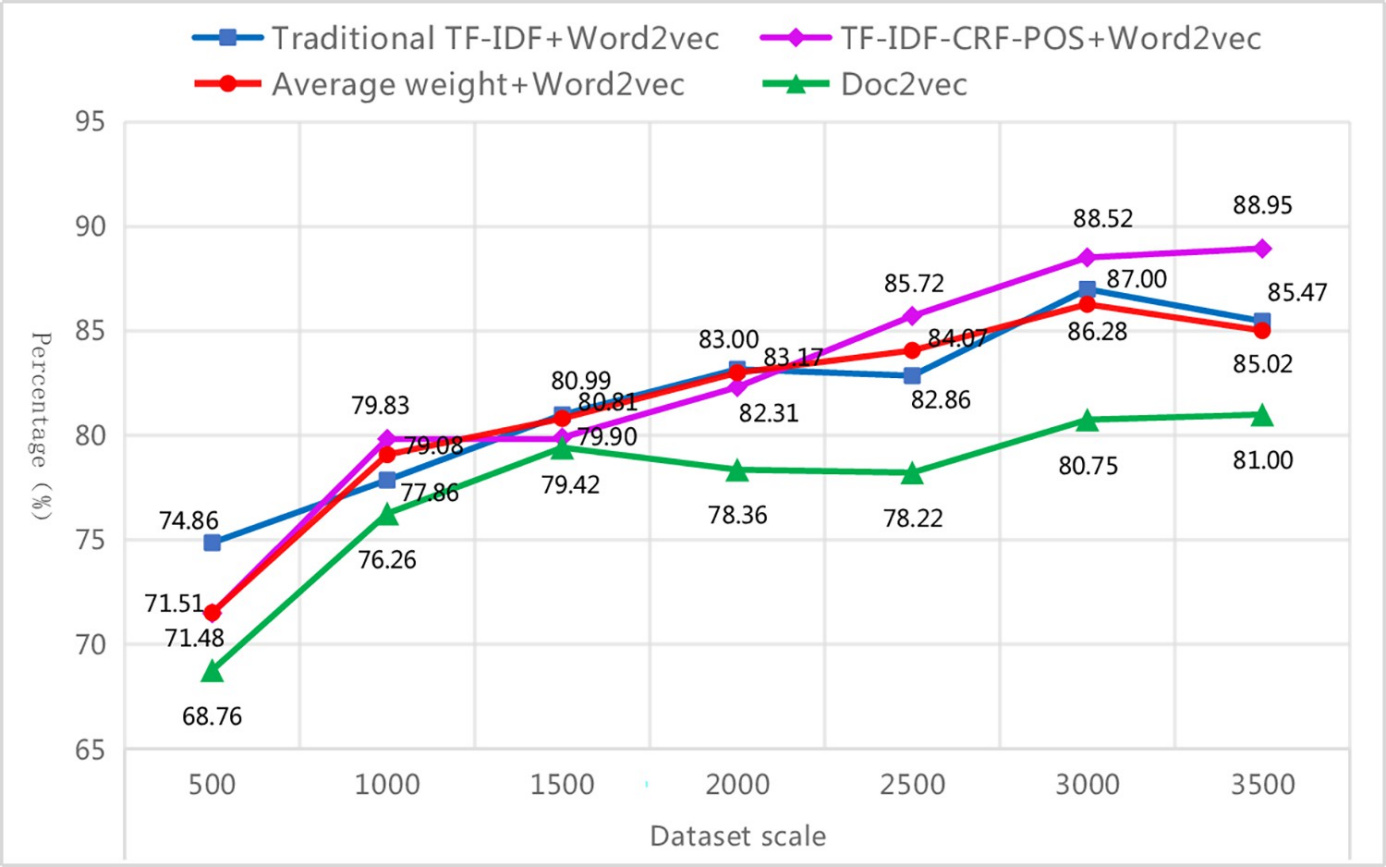
Weighted-F1 of the optimal combination model with different scale text sets.

The improved method in this paper can steadily improve the classification performance, reaching a high and stable level with the increase of training scale. To verify the stability of the improved method, text sets of different scales of 500, 1000, 1500, 2000, 2500, 3000, and 3498 respectively were selected for this experiment. The text sets were uniformly divided into training and test sets in a 4:1 ratio. The optimal weighted-F1 of each text representation method was used as the criterion to determine the best combination model under different scale text sets, thereby comparing the superiority and inferiority of each text representation method under different scale training and test sets (as shown in Table 11 and Fig 11). From Table 11, it can be observed that the top three matching classifiers for the four text representation methods under different scale text sets are MLP, SVM, and LR, which is consistent with the conclusions obtained above. Specifically, the traditional TF-IDF+Word2Vec method is more suitable when matched with SVM, the improved method is more suitable when matched with MLP, the average weight+Word2Vec method shows no obvious preference for any particular classifier, and the Doc2vec method is more suitable when matched with LR. With the change of text set scale, the results indicate that KNN and NB are more suitable for smaller scale text sets, SVM is more suitable for medium scale text sets, and MLP and LR are more suitable for both smaller and larger scale text sets. Fig 11 shows that as the scale of the text set increases from 500 to 3500, the weighted-F1 of the best combination model under each text representation method increases, but their growth rate and fluctuation trend are not the same. Overall, the classification performance of the proposed method is optimal in all other scale text sets, except for the medium scale (1500 and 2000) and small scale (500) text sets, where it is slightly lower than the traditional TF-IDF+Word2Vec method and average weight +Word2Vec method. And it steadily improves with the increase of text set scale and reaches a high and stable point.

**Table 11:** The optimal combination of text representations and classifiers for different-size text sets.

Text set size Best match classifier Text representation method	Traditional TF-IDF +Word2Vec	TF-IDF-CRF-POS +Word2Vec	Average weight +Word2Vec	Doc2vec
500	*+KNN*	+KNN	+NB	+NB
1000	*+SVM*	+LR	+MLP	*+LR*
1500	+SVM	+SVM	+SVM	+LR
2000	+SVM	+SVM	+SVM	+LR
2500	*+MLP*	+MLP	*+MLP*	+MLP
3000	+LR	+MLP	*+LR*	+SVM
3498	+MLP	+MLP	+LR	+LR
Total	*3SVM+2MLP+LR+KNN*	3MLP+2SVM+LR+KNN	2MLP+2SVM+2LR+NB	4LR+SVM+MLP+NB

**Fig 12:**
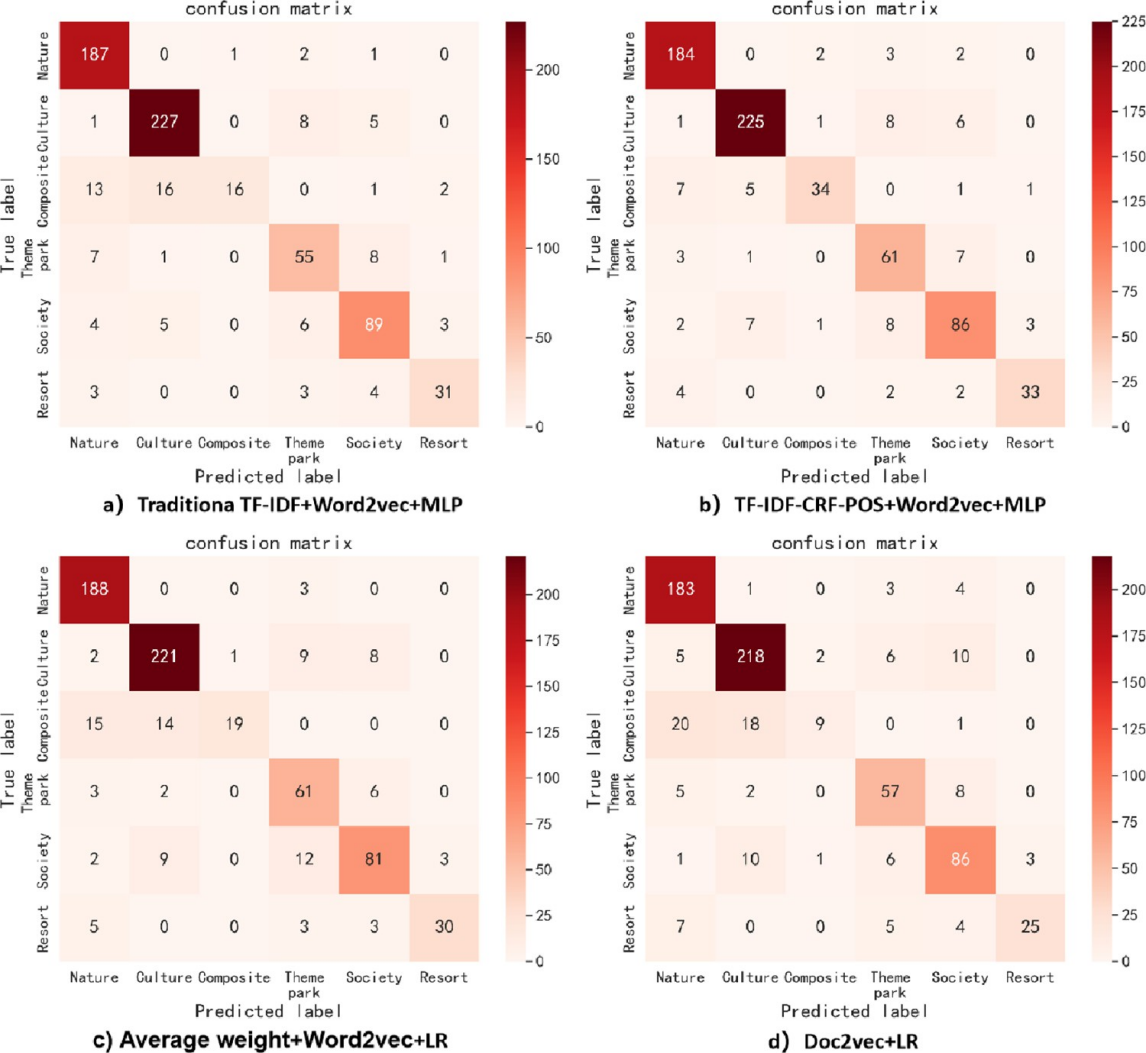
Confusion matrix heat map of optimum classification combination models under text set size of 3498.

**Fig 13:**
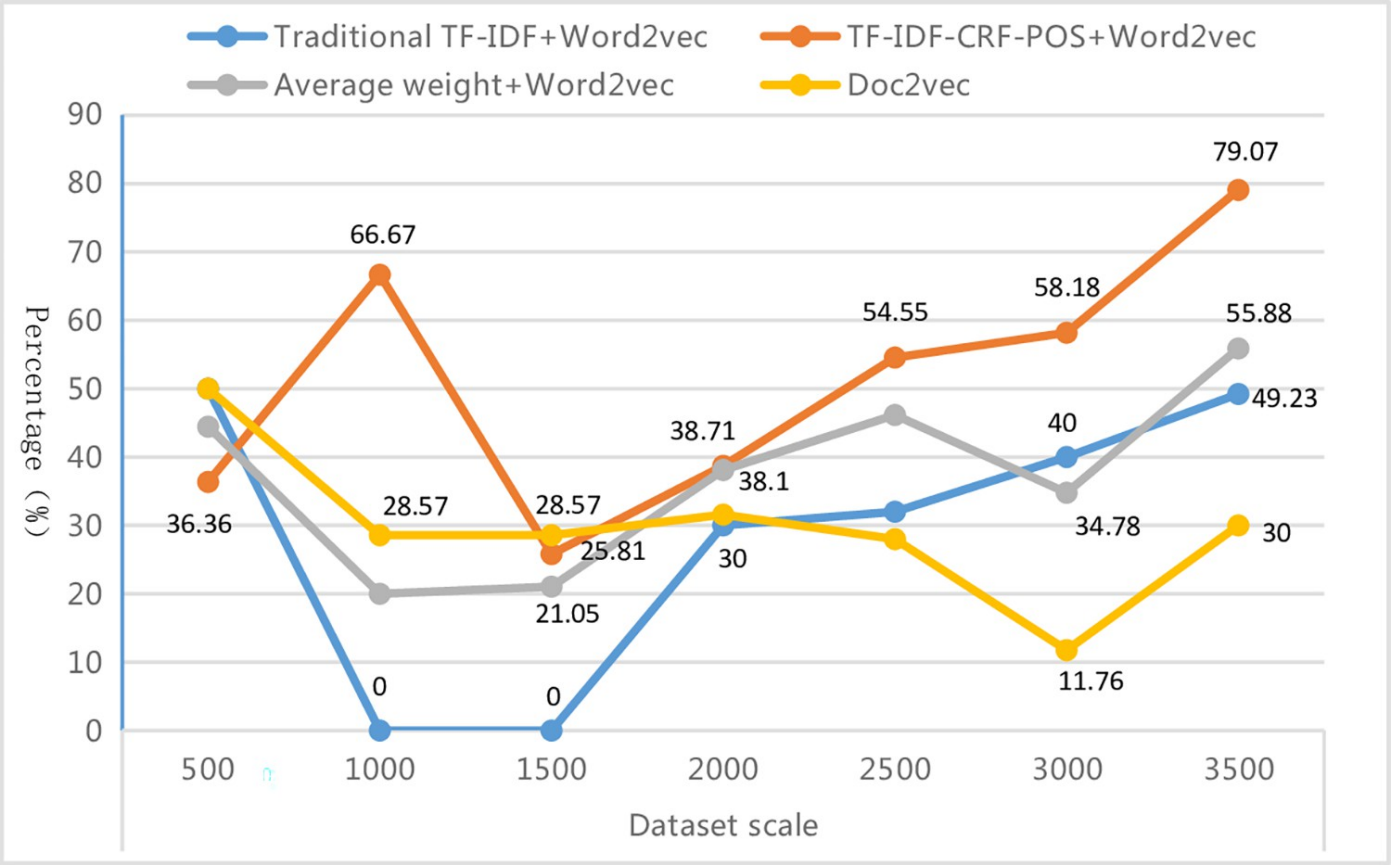
F1-measure of the composite category of optimum classification combination models under different scale text sets.

The ability of the proposed method in this paper to identify all categories of the imbalanced datasets across all scales of text sets indicates that its robustness is the best. Although the composite index value "weighted-F1" of this proposed method is slightly lower than that of other methods at some scales, its recognition performance for various specific categories is more balanced and stable (as shown in Fig 12), and it can effectively handle rare categories with low support. Particularly in the composite category with the minimum number of samples (as shown in Fig 13), it outperforms other methods. For example, at scales above 1,000, the proposed method achieves better identification performance for the composite category compared to the traditional TF-IDF+Word2Vec method and average weight+Word2Vec method. Even at the scales of 1,000 and 1,500, the recognition performance of the traditional TF-IDF+Word2Vec method for this category is 0%, meaning that no samples of this category are recognized. However, during the same scales, the proposed method achieves recognition performance of 66.67% and 25.81% respectively for the composite category. For classification tasks such as tourist attractions, the algorithm results should not be in favor of any specific category, because the recognition of rare categories in tourist attractions is equally important. Thus, the significance of this proposed method in the classification of tourist attractions is highlighted.

#### 4.4.4. Comparative analysis of application results

**Fig 14:**
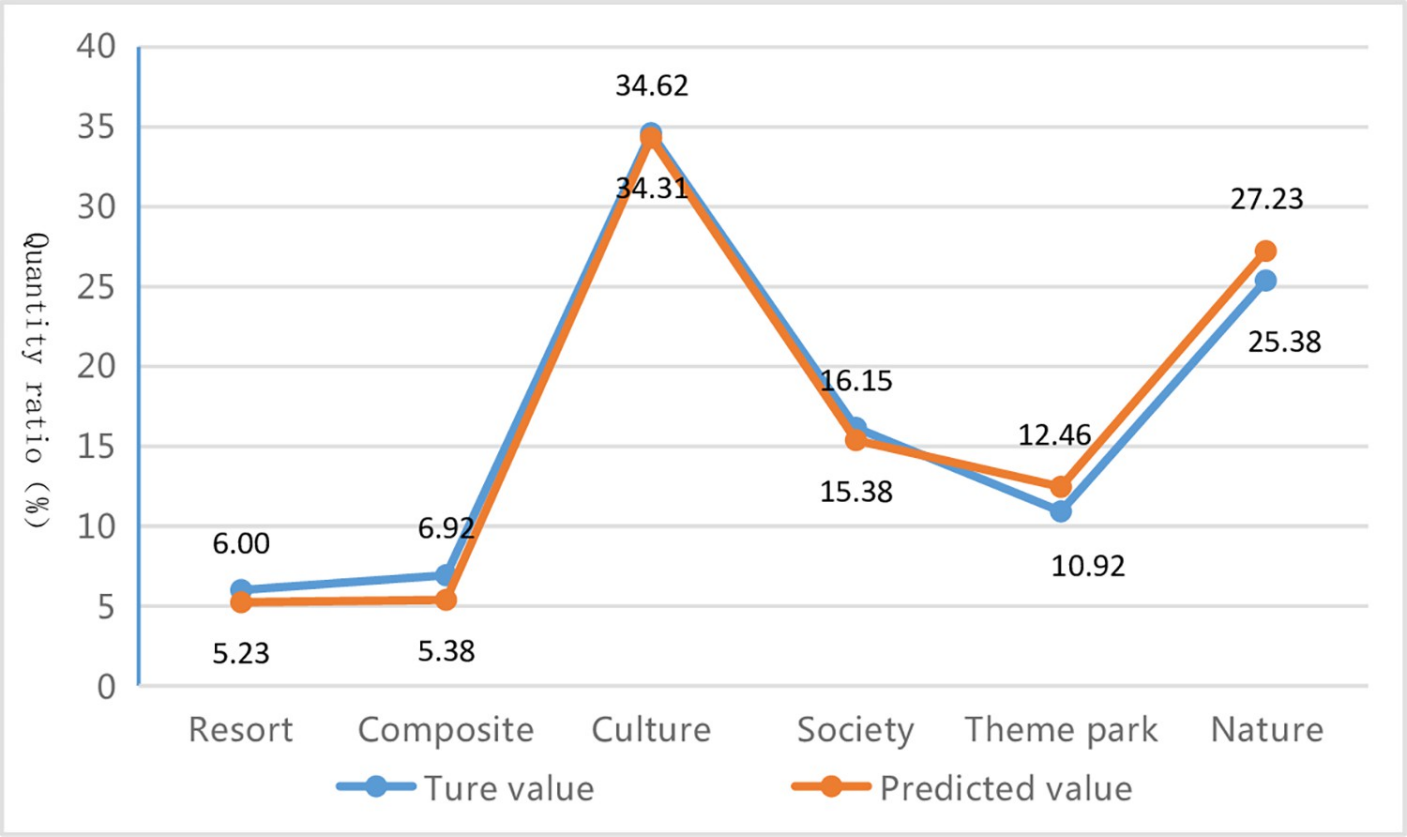
Quantity ratio of the true and predicted values for each category of tourist attractions.

**Fig 15:**
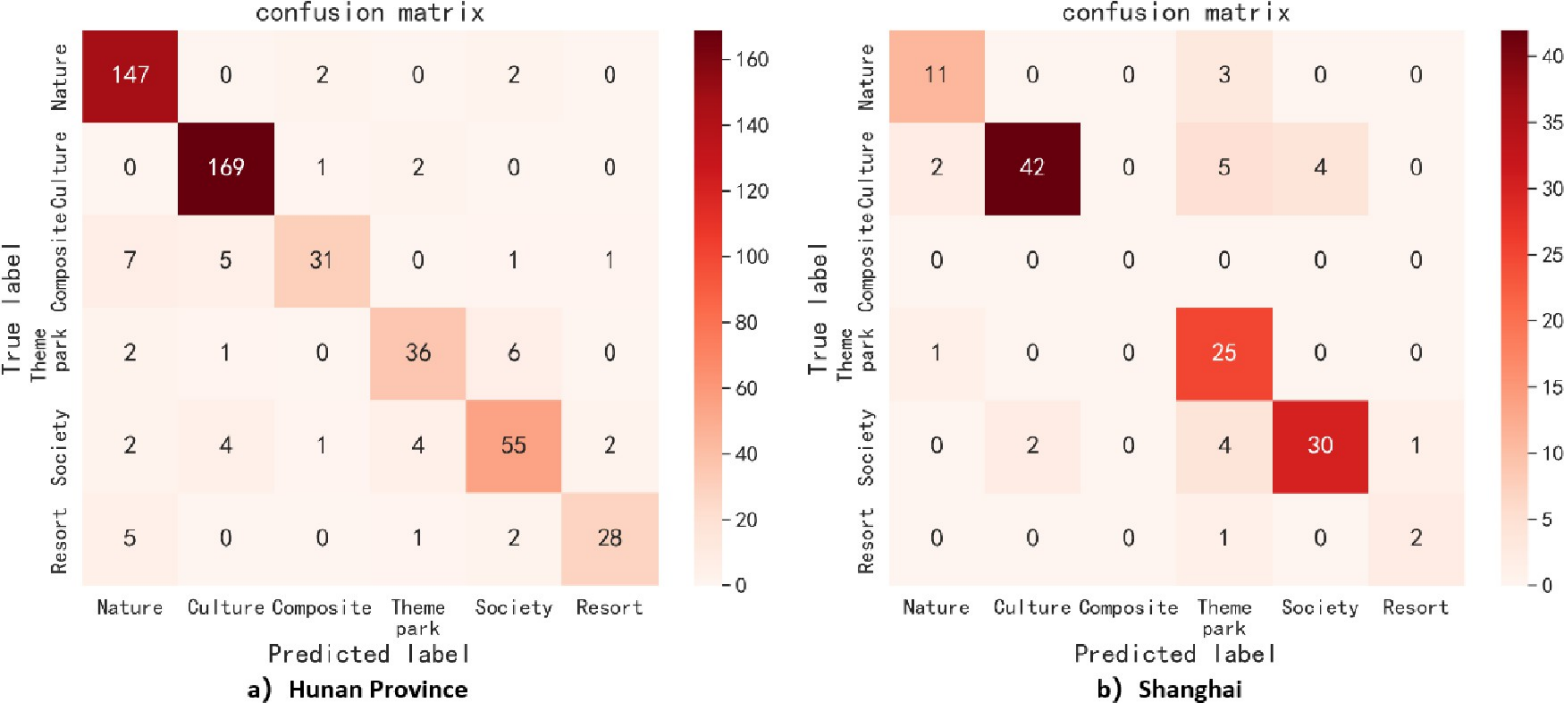
Confusion matrix heat map of the test set in Shanghai and Hunan Province.

To ensure the overall integrity of the application result analysis, a new test set consisting of 650 description texts from two provincial-level regions, Hunan Province and Shanghai City, was selected. The improved method (TF-IDF-CRF-POS +Word2Vec+MLP algorithm) was used to predict the new test set, and the predicted classification values were compared with the true values. The comparisons are shown in Tables 12–14 and Figs 14 and 15.

**Table 12:** Comparing true and predicted values of the top 2–3 categories in different level attractions.

Scenic spot level	Counting	Percentage	The top 2–3 categories	
			Result of true value	Result of predicted value
1A	2	0.31%	Culture, Nature	Culture, Nature
2A	29	4.46%	Nature, Culture	Nature, Culture
3A	397	61.08%	Culture, Nature, Society	Culture, Nature, Society
4A	207	31.85%	Culture, Nature, Theme park	Culture, Nature, Theme park
5A	15	2.31%	Nature, Culture	Nature, Culture

**Table 13:** Quantity ratio of the true and predicted values of various attractions in the two provinces.

Province or city	Category	True value count	Percentage	Predicted value count	Percentage	Province or city	Category	True value count	Percentage	Predicted value count	Percentage
Hunan Province (517)	Resort	36	6.96%	31	6.00%	Shanghai City (133)	Resort	3	2.26%	3	2.26%
	Composite	45	8.70%	35	6.77%		Composite	0	0.00%	0	0.00%
	Culture	172	**33.27%**	179	**34.62%**		Culture	53	**39.85%**	**44**	**33.08%**
	Society	68	**13.15%**	66	**12.77%**		Society	37	***27*.*82%***	***34***	***25*.*56%***
	Theme Park	45	8.70%	43	8.32%		Theme Park	26	***19*.*55%***	***38***	***28*.*57%***
	Nature	151	**29.21%**	163	**31.53%**		Nature	14	10.53%	14	10.53%

**Table 14:** Comparing true-predicted values of the top 2–3 categories in different-level attractions in the two provinces.

Province	Scenic spot level	Counting	Percentage	The top 2–3 categories	
				Result of true value	Result of predicted value
Hunan Province	1A	2	0.39%	Culture, Nature	Culture, Nature
	2A	29	5.61%	Nature, Culture	Nature, Culture
	3A	336	64.99%	Culture, Nature, Society	Culture, Nature, Society
	4A	139	26.89%	Culture, Nature, Theme park	Culture, Nature, Theme park
	5A	11	2.13%	Nature, **Composite, Culture**	Nature, **Culture, Composite**
Shanghai	3A	61	45.86%	Society Culture, Theme Park	Society, Culture, Theme Park
	4A	68	51.13%	Culture, Theme Park, Society	Culture, Theme Park, Society
	5A	4	3.01%	Culture, Theme Park	Culture, Theme Park

#### Firstly, it is the comparison of the summary situation

From Fig 14, it can be found that there is minimal difference between the true and predicted proportions of each category of tourist attractions and the trend curve is largely similar. The proportion of tourist attractions can be ranked in the following order: culture, nature, society, theme park, composite, and resort categories. The largest difference was found in the nature category, but it was only 1.85%. Table 12 shows that the top 2–3 categories having the highest counts in different levels of tourist attractions, both in terms of true and predicted values, are completely consistent. Additionally, in each level, the top two categories with the largest number of tourist attractions are cultural and natural categories.

#### Secondly, it is the comparison of different provinces

As shown in Table 13, the distribution tendencies of scenic spot categories differ among different provinces. For example, under the true value, the top three categories of tourist attractions in Hunan Province, in terms of quantity, are culture, nature, and society categories, whereas, in Shanghai, the top three categories are culture, society, and theme park categories. In terms of the difference between the true value and predicted value, Shanghai shows a larger difference than Hunan Province. The confusion matrix heat map, Fig 15, reveals that this difference is due to the smaller sample size of various tourist attractions in Shanghai compared to Hunan Province, which can result in larger prediction deviations. Some of the cultural tourist attractions in Shanghai (5 out of 53) are predicted to be theme parks, and the predicted value of the theme park category exceeds the true value. Therefore, according to the final predicted values, the top three categories of tourist attractions in Shanghai are culture, theme park, and society categories. Although this predicted result is slightly different from the true value conclusion, it still falls within an acceptable range. By further subdividing and comparing the true value with the predicted value of various tourist attractions at different levels in Hunan Province and Shanghai, as shown in Table 14, it is found that the ranking of the top 2–3 categories based on quantity ratio is almost the same between the true and predicted values, except for the ranks of composite and culture categories of 5A-level tourist attractions in Hunan Province. Upon closer examination, it was discovered that the number of 5A-level tourist attractions in Hunan Province is relatively small (only 11) and the total number of composite and culture categories of tourist attractions is only 5. The improved method has a prediction error of 2. The reason for the difference in sorting is that under the circumstance of a limited number of predicted samples, the randomness of sample selection results in the composite category and the culture category, which already have a high similarity, coincidentally encountering indistinguishable similar samples, leading to significant errors. However, as the number of prediction samples increases, the error caused by the randomness of sample selection will decrease. Therefore, the 60% accuracy here is acceptable.

In conclusion, the overall accuracy of the prediction results in using the improved method exceeds 88%. The predicted value aligns closely with the true value, with minimal deviation that is within a controllable and acceptable range. Therefore, the improved method is considered feasible and effective, and it can be applied to the actual prediction of texts with similar content in the tourist attraction field and other similar fields.

## 5. Conclusion

Most of the existing text classification research is based on English datasets, and research on Chinese text datasets is still relatively limited, primarily relying on commonly used public corpora. Although these corpora have high quality, they are from an earlier period, such as the datasets from Sogou Lab and THUC News, which are all from before 2012, and they have significant differences from domain-specific corpora. This means that algorithms that perform well on these public Chinese corpora may not be suitable for classifying domain-specific language data. The method explored in this paper aims to achieve automatic classification of Chinese text corpora in specialized fields, such as tourist attractions, where the complexity of text types and similarity of content in domain-specific corpus make classification more challenging. The type of text dataset used in this paper is quite complex, as shown in the following aspects: First, it includes texts of varying lengths, ranging from 28 to 440 characters, which involve both short and medium-length texts and is relatively complex. Short texts typically have fewer words compared to longer texts, making it difficult to extract their features within a limited word count. This results in insufficient feature representation and affects the accuracy of Chinese short-text classification. Second, the number of effective samples for different categories in the text dataset varies significantly, that belongs to an imbalanced dataset where some categories have strong similarities, such as culture and complex categories, and even manual discrimination has certain difficulties. Similar text contents of subcategories indicate that there are strong semantic relations among subordinate categories of the same main category, the words within the texts are highly similar, and the distinction between categories is small. Multiclass automatic classification research on this type of text is more challenging than classification research with obvious category distinctions, and relevant research is scarce at present, so further exploration is needed. Therefore, this paper focuses on the algorithmic research of automatic classification of imbalanced Chinese text datasets in the domain-specific field with multiple types of text lengths and strong similarity categories. It can effectively achieve the multi-classification of content-similar subcategories. This is a valuable exploration of applying algorithms to complex Chinese text datasets. The findings can not only be used for the classification of tourist attraction description texts in the tourism field but also for the classification of similar texts in other aspects of the tourism field or other professional fields, as well as in other fields such as scientific research and education that require sub-category classification.

Moreover, the improvement made in this paper is more comprehensive than those considered in existing research, and the effectiveness of the improved model has been demonstrated through detailed experimental processes from the improvement process, overall model performance, stability, and application conclusions. The experiment showed that the improved model can achieve good results when combined with the MLP classifier, and its overall accuracy, precision, recall, and F1-measure all reach approximately 89%. Its performance is even better than that of the BERT classification model,which has achieved excellent performance in extensive tasks, and is respectively higher by 2.29%, 5.55%, and 2.90% in Acc, marco-F1, and mirco-F1 values. Furthermore, the model can identify rare categories well, especially for the identification of composite category with the poorest classification performance. The F1-measure on the composite category is 23.19%~49.07% higher than that of other algorithms. The considerable difference in marco-F1 values between the aforementioned BERT model and the improved method in this paper is also due to the BERT model’s much lower performance on rare categories compared to this improves method. The above statements indicate the effectiveness of this improved method. In addition, the overall performance of the improved method and the recognition performance of rare categories on text sets of different sizes have always been at the forefront. With the increase of the text set size, the classification performance can steadily improve to reach a high point of stability, indicating that the improved method has good robustness. Finally, when the improved method was applied to prediction, the conclusions obtained from the true value and the predicted value were basically consistent. The conclusions are that “the most common categories were culture and nature, and the tendency of tourist attraction categories varied among different provinces”. It indicates that the improved method is practical and feasible.

In terms of data collection, only a portion of the descriptive texts of tourist attractions (approximately 1/4) is selected as experimental samples in this paper. This is mainly due to the following considerations: Firstly, there are some limitations in the data collection, which are mainly limited by the fact that "the collection of all the tourist attractions’ texts will cost a lot of manpower and time because of the inconsistency of data sources"; Secondly, the core objective of this study is not to collect all the texts of tourist attractions, but rather to verify the feasibility of the proposed algorithm and its effectiveness in practical application. Therefore, we argue that even if all the introductory texts of tourist attractions are not collected, the method proposed in this paper still has practical application value. For instance, when a user wants to know what classification a certain tourist attraction belongs to, they only need to input the introductory text of that tourist attraction into the model of this paper, and then they can obtain the classification result. Furthermore, to maintain the randomness of the samples and their completeness in the provinces, this paper randomly selected a comprehensive set of tourist attraction from several provinces as the research samples to collect the introduction texts. This strategy not only meets the research objective of this paper but also takes into account the actual situation.

Additionally, although TF-IDF is an old concept proposed earlier, it continues to demonstrate strong practicality and broad application prospects in current research across various fields. For example, in the field of information retrieval [41], TF-IDF can effectively assess the relevance between query terms and documents, thereby assisting in determining and providing the most relevant search results; in the task of sentiment analysis [42], TF-IDF can rank the importance of each keyword in various types of sentiment polarity texts, which aids in a deeper understanding of user needs and focal points; in the area of keyword extraction [43], TF-IDF can identify and extract core keywords in documents, assisting with tasks such as document summarization and information indexing; for topic discovery [44], researchers can reveal underlying topics or trends by analyzing the words with higher TF-IDF values in a set of documents; in social media analysis [45], TF-IDF has also been used to identify trending topics and influential users. Therefore, this paper focuses on the improvement of TF-IDF and combines it with Word2Vec to study text classification tasks, which still have important theoretical and practical value.

Although the improved model in this paper has good performance and robustness, it still needs to be further improved in some aspects: First, the text corpus used in the experiment is limited to Chinese, and it has yet to be proven whether this method is equally effective for English and other languages. Second, when using this method for other professional fields, it still requires adjustments according to the actual corpus, such as adjusting the custom dictionary and stop word list. Third, the object of this paper is subcategories classification task with similar content, which has high requirements on the corpus set. Existing similar text datasets have not been found for verification, and due to limited human resources and time, this paper’s data collection is limited to the descriptive texts of tourist attractions. In the future, more text sets from other aspects of the tourism industry or other professional fields can be collected and used to verify and perfect the improved method. Additionally, the improved model in this paper can be integrated with deep neural network models to explore whether higher accuracy can be achieved.

## Supporting information

S1 File Customized dictionary. (TXT)

S2 File Customized stop words list. (TXT)

S3 File Tourist attraction description text data. (TXT)

S4 File Tourist attraction classification data set. (XLS)
